# The effects of robotic assistance on upper limb spatial muscle synergies in healthy people during planar upper-limb training

**DOI:** 10.1371/journal.pone.0272813

**Published:** 2022-08-08

**Authors:** Adriana Cancrini, Paolo Baitelli, Matteo Lavit Nicora, Matteo Malosio, Alessandra Pedrocchi, Alessandro Scano

**Affiliations:** 1 Department of Electronics, Information and Bioengineering, Neuroengineering and Medical Robotics Laboratory, Politecnico di Milano, Milan, Italy; 2 Department of Neuromotor Physiology, Laboratory of Visuomotor Control and Gravitational Physiology, Fondazione Santa Lucia, IRCCS, Rome, Italy; 3 UOS STIIMA Lecco - Human-Centered, Smart & Safe, Living Environment, Italian National Research Council (CNR), Lecco, Italy; 4 Industrial Engineering Department, University of Bologna, Bologna, Italy; University of Pittsburgh, UNITED STATES

## Abstract

**Background:**

Robotic rehabilitation is a commonly adopted technique used to restore motor functionality of neurological patients. However, despite promising results were achieved, the effects of human-robot interaction on human motor control and the recovery mechanisms induced with robot assistance can be further investigated even on healthy subjects before translating to clinical practice. In this study, we adopt a standard paradigm for upper-limb rehabilitation (a planar device with assistive control) with linear and challenging curvilinear trajectories to investigate the effect of the assistance in human-robot interaction in healthy people.

**Methods:**

Ten healthy subjects were instructed to perform a large set of radial and curvilinear movements in two interaction modes: 1) free movement (subjects hold the robot handle with no assistance) and 2) assisted movement (with a force tunnel assistance paradigm). Kinematics and EMGs from representative upper-limb muscles were recorded to extract phasic muscle synergies. The free and assisted interaction modes were compared assessing the level of assistance, error, and muscle synergy comparison between the two interaction modes.

**Results:**

It was found that in free movement error magnitude is higher than with assistance, proving that task complexity required assistance also on healthy controls. Moreover, curvilinear tasks require more assistance than standard radial paths and error is higher. Interestingly, while assistance improved task performance, we found only a slight modification of phasic synergies when comparing assisted and free movement.

**Conclusions:**

We found that on healthy people, the effect of assistance was significant on task performance, but limited on muscle synergies. The findings of this study can find applications for assessing human-robot interaction and to design training to maximize motor recovery.

## Introduction

Cerebral stroke is one of the main causes of mortality which ranks second worldwide and third in Italy, causing about 10% of deaths per year [1, 2]. Considering the disability-adjusted life year (DALY), which expresses the number of years lost due to a specific disease, it emerges that stroke holds the primacy among the causes of disability (35% of patients). People affected by stroke may present motor deficits that compromise their lives and autonomy. Robotic devices for neurorehabilitation have been introduced in the market to offer a valid alternative to conventional therapy and to fill the constantly growing gap between supply and demand [3, 4]. Since the pioneering approaches in the 90s [5], in the last decades, several studies [6, 7] showed that robotic rehabilitation is an effective technique inducing comparable or better motor improvements in respect to standard treatment [8], often including bioinspired control [9]. However, the mechanisms underlying motor recovery and neuroplastic effects induced by robotic therapy are not well known and understood yet. It is in fact a matter of debate which guidelines should be followed when assisting rehabilitation with robots. While several good practice guidelines have been proposed, including: assist-as-needed paradigms [10], adoption of transparent robots [11], biomimetic controllers [12], human-in-the-loop approaches [13], adaptive controllers [14], some relevant scientific questions are still open. In particular, detailed evidence on the effect of robot assistance on motor control have not been exhaustively identified [15, 16]. Muscle synergies (MS) are a quantitative framework for evaluating motor coordination [17]. According to MS, the CNS exploits a reduced set of pre-shaped neural pathways, called synergies, to achieve a large variety of motor commands. The potential of this method was exploited to gain deeper insights concerning motor impairment. Cheung and colleagues [18, 19] suggested that in post-stroke patients, spatial synergies are preserved even if their recruitment timing is altered and they also found that in severely impaired patients, effects such as fractionation or merging of synergies might be used as biomarkers for assessing motor control alterations. Interestingly, MS have also been used as metrics for the evaluation of robot-assisted interventions [20], finding that post-stroke subjects who followed robotic rehabilitation showed larger improvements in axial-to-proximal muscle synergies with respect to those who underwent usual care. This was associated with a significant improvement of the proximal kinematics, but with negative effects in muscle synergies controlling the distal body segments. It was also shown that robot-therapy induced subject-dependent modification of synergies [21] and slight modifications of the original synergies [22]. In our literature analysis, we noted that only a few studies have evaluated the effects of assistance and challenging conditions during human-robot interaction on healthy people. In our view, this assessment is a missing piece in the understanding of how robot assistance influences motor coordination and for the identification of which modes and approaches can maximize patients’ motor recovery. Following this rationale, the aim of our work is to investigate the effect on muscle synergies of human-robot interaction on healthy people when the robot provides assistance, and to determine how MS are altered when assistance intervenes. These achievements will be a preliminary step to fill the gap that still exists between the availability of robot operators and the guideline principles that should regulate their best use in clinical scenarios, eventually exploiting their potential toward therapy personalization.

## Materials and methods

**Fig 1:**
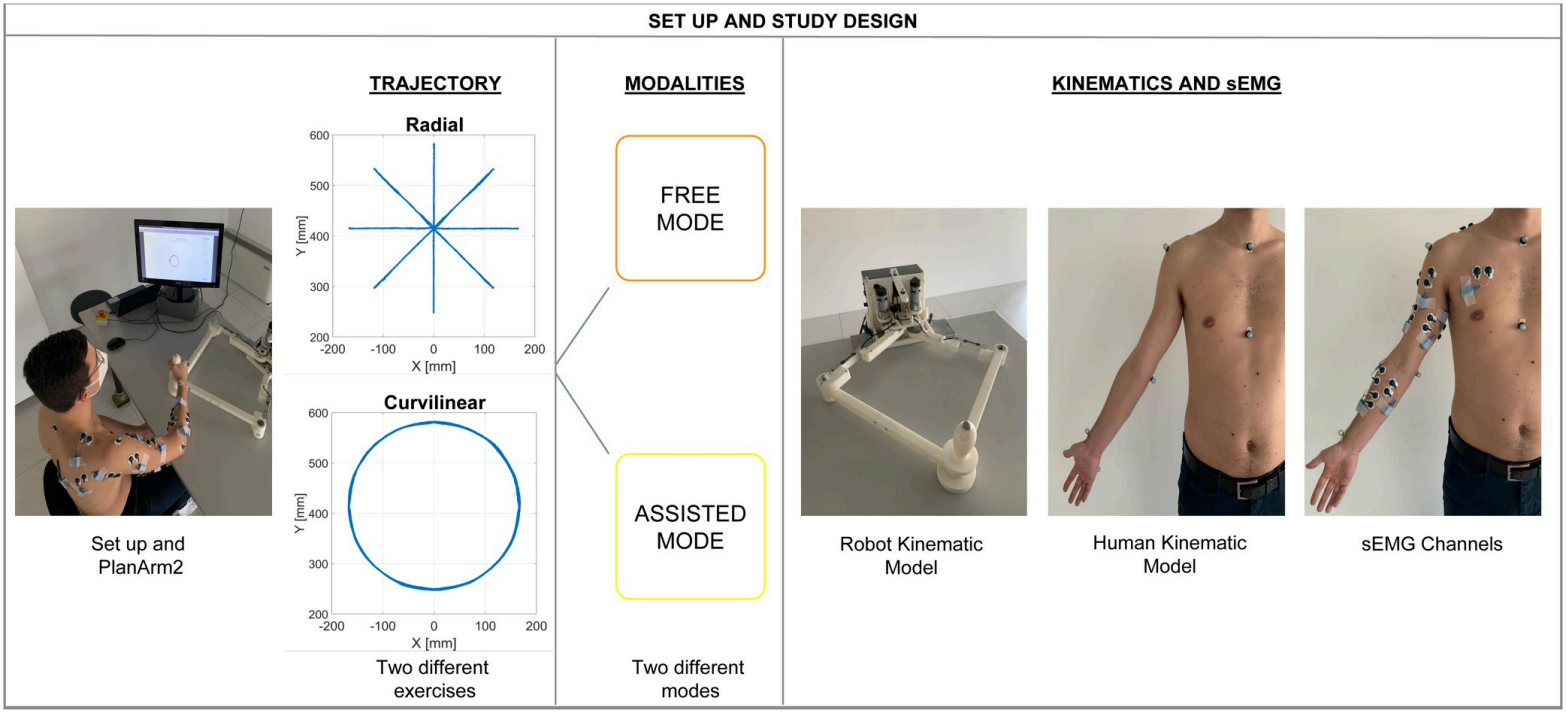
Study design. The left side of the scheme describes the experimental protocol, that included: 2 exercises with point-to-point movements (Radial) and curvilinear movements (Curvilinear), carried out in 2 different modes (Free and Assisted) repeated each one 10 times (total of 40 repetitions). The right panel shows the kinematic experimental models and considered signals. The aim of the work is to investigate muscle synergies of the upper-limb in a context of human-interaction to characterize the effects of robot assistance. Tracking data was elaborated with the Vicon system and EMG with a 16 channel wireless system.

An overview of the study design is portrayed in Fig 1.

### Experimental set-up

**Fig 2:**
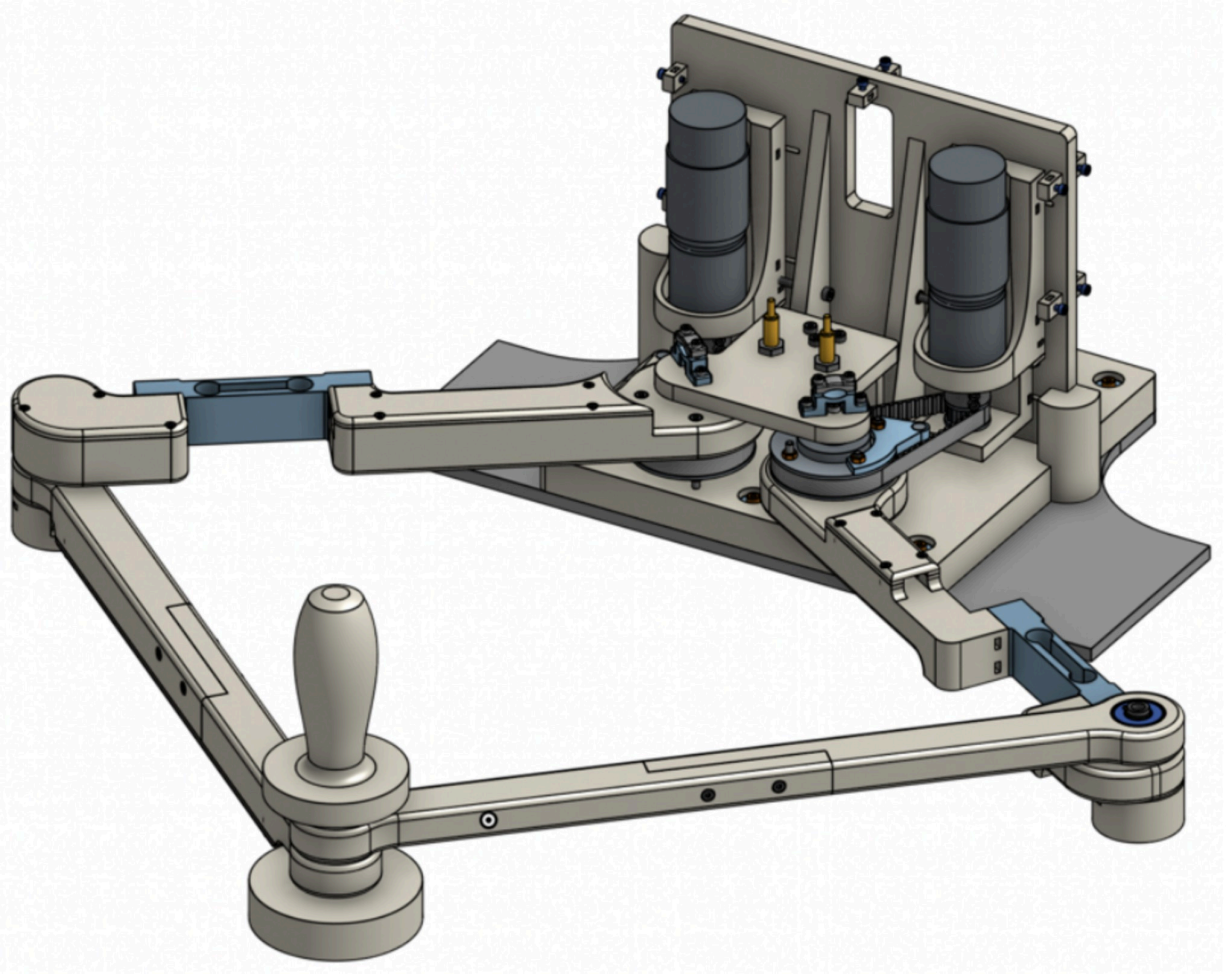
The PlanArm2 prototype.

*Robot*—The robot used for the experimental trial, represented in Fig 2, is the PlanArm2 prototype [23], a planar robotic platform developed within CNR specifically for upper-limb training and available at the CNR STIIMA laboratories (Lecco, Italy). This device is characterized by a parallel architecture realizing two degrees of freedom on the horizontal plane and equipped with two DC motors and two load cells that enable the implementation of force-based control algorithms with the subject at the end-effector level.

#### Control

The control system of PlanArm2 runs within the ros_control framework, a flexible architecture that enables simple implementation and deployment of robot controllers [24].

The robot control had already been implemented prior to this study. In particular, for this specific study, the following control strategies have been selected:

1. *Position control for the execution of ideal trajectories (Reference adaptation)*: Key waypoints are taken as input and parameterized into a complete trajectory with trapezoidal speed profile. The end-effector is moved along the computed ideal trajectory by the motors while the subject must follow it as precisely as possible, minimizing the force exchanged between the hand and the manipulandum.
2. *Admittance control for the minimization of interaction forces (Free)*: A 0N force tracking logic is used to minimize the force required to freely move the end-effector within the workspace of the robot. No assistance is provided in this case; the goal is to render the device as transparent as possible and let the user perform the task independently.
3. *Tunnel control for subject assistance (Assisted)*: The previous controller is expanded to assist the subject in performing the task. Given an ideal path, a virtual tunnel of predefined width is constructed around it. As long as the end-effector is kept within those boundaries, interaction forces are minimized in order to render the device as transparent as possible. Instead, if the end-effector is pushed outside of the tunnel, a restoring force is produced to correct the subject’s motion and provide the required assistance.

#### GUI

Finally, the system is completed by a graphical user interface developed in Unity3D and directly communicating with the control system of the robot. The GUI provides the user with visual feedback on the task to be performed (targets and paths).

#### Sensors

During the trials, subjects were approximately in the middle of the area tracked by the motion capture system (Vicon 10 TVC system, Oxford, UK), sitting on a chair in front of a table equipped with the PlanArm2 robot and a screen. During the trials, subjects wore a set of eleven markers, positioned on T8 and C7 vertebrae, jugular notch, xiphoid process, greater tubercle, medial and lateral humeral epicondyles, styloid process of the ulna and the radial, second and fifth metacarpal heads. The protocol was inspired from Dumas et al. [25]. Markers were placed also on the robot to track the movement (a set of six markers, three of these positioned on the base to represent the reference axes; one on the top of the right driven pulley; one on the right joint and the last one on the end-effector). The recordings were made with the Vicon System (Oxford, United Kingdom). Subjects were instrumented with 16 s-EMG electrodes (Cometa, Italy) positioned according to the SENIAM guidelines [26] on the following muscles: Erector Spinae (ES), Middle Trapezius (MT), Upper Trapezius (UT), Infraspinatus (IF), Deltoid Anterior (DA), Deltoid Middle (DM), Deltoid Posterior (DP), Pectoralis (PC), Triceps Long Head (TLo), Triceps Lateral Head (TLa), Biceps Long Head (BCl), Biceps Short Head (BCs), Brachioradialis (BR), Pronator Teres (PT), Wrist Flexors (WF) and Wrist Extensors (WE).

### Experimental protocol

The study took place at the Consiglio Nazionale delle Ricerche (CNR—Italy), UOS Lecco, Human Motion Analysis Laboratory. The study was reviewed and approved by the Politecnico di Milano Ethical Committee. All subjects gave consent to participate and signed a written informed consent before the experiment, which was conducted in accordance with the Declaration of Helsinki.

In this study, 10 healthy adult individuals, neurologically and orthopedically intact, were recruited. We investigated two main conditions while in interaction with the robot; they were preceded by a short adaptation phase to familiarize with the task to be executed. During preliminary adaptation, the robot was moved with a position controller from target to target with a rectilinear or curvilinear path and trapezoidal-shaped biomimetic velocity profile. Each subject was asked to follow the robot without pushing nor being pushed, thus keeping interaction forces as low as possible.

The first experimental condition that we considered was “Free”. In this condition, the robot controller was set to increase at maximum robot compliance, resulting transparent to the user. Each subject had to reproduce the trajectories performed in the Reference condition without any assistance. Participants were asked to reproduce exactly the trajectories shown on the screen. During the movement, the display showed the starting point, the end point, the ideal spatial profile and the trajectory carried out by the subject. As a kinematic metric for task performance, we measured the root mean error for each of the samples of the performed trajectory in respect to the requested one.

The second experimental condition that we considered was “Assisted” in which subjects had to move the end-effector of the robot as in the Free mode, but with an assistance tunnel with 10 mm width.

**Fig 3:**
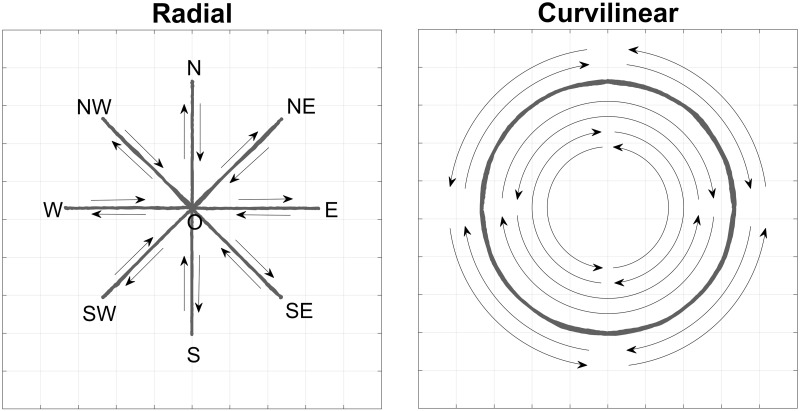
A schematic representation of exercises trajectories: Radial (left) and curvilinear (right).

This measure was set after pre-trial tuning of the assistance force; therefore, the tunnel was not rigid but soft, and it did not completely prevent crossing the tunnel boundaries. The subjects performed the entire protocol using the dominant limb. The acquisition protocol included a comprehensive variety of movement trajectories, based on standard radial paradigms (circular multidirectional targets) [5, 21] and curvilinear trajectories (Fig 3). While radial paths are a standard for this set-up, curvilinear tasks were only marginally considered in the literature of robot-assisted planar movements. We decided to implement such tasks to elicit a wide variety of upper-limb tasks to promote challenge and the recruitment of the synergies available to people [27]. The targets were oriented toward the main cardinal directions (NE, E, SE, S, SW, W, NW, N) in a circumference with a radius of 170 mm. This dimension was comparable to previous studies [21].

The first considered condition was Radial, characterized by radial movements. These movements began with the hand in the center of each target set and consisted in going towards each of the peripheral targets and then coming back to the center target (O-NE, NE-O, O-E, …). Each acquisition trial was composed of 16 movements (reaching 8 targets and coming back). After each movement, the subject had to wait for about one second before proceeding to the next target. Subjects were required to move slightly fast, in order to enhance the EMG related to phasic (dynamic) EMG activity. Following this instruction, subjects were expected to complete Radial exercises in no more than 40–45 s. However, tolerance in execution time was accepted. To prevent fatigue, a 30-second pause was introduced after each trial if the subject needed it.

The second task was Curvilinear. Cardinal points were used as the beginning and end of the curvilinear trajectories, and as way-points. In particular, subjects performed alternately ¼ circle and ½ circle, first clockwise and then anticlockwise (e.g.: from south to west, passing through south-west: S-SW-W; from west to east, passing through north: W-N-E;…). Following this instruction, subjects were expected to complete Curvilinear exercises in no more than 50–55 s. For each condition, all the tasks were repeated 10 times as in similar protocols [28]. The protocol had a duration of about 1h 30 min/2 hours per subject, including the sensor dressing phase.

### Data analysis methods

**Fig 4:**
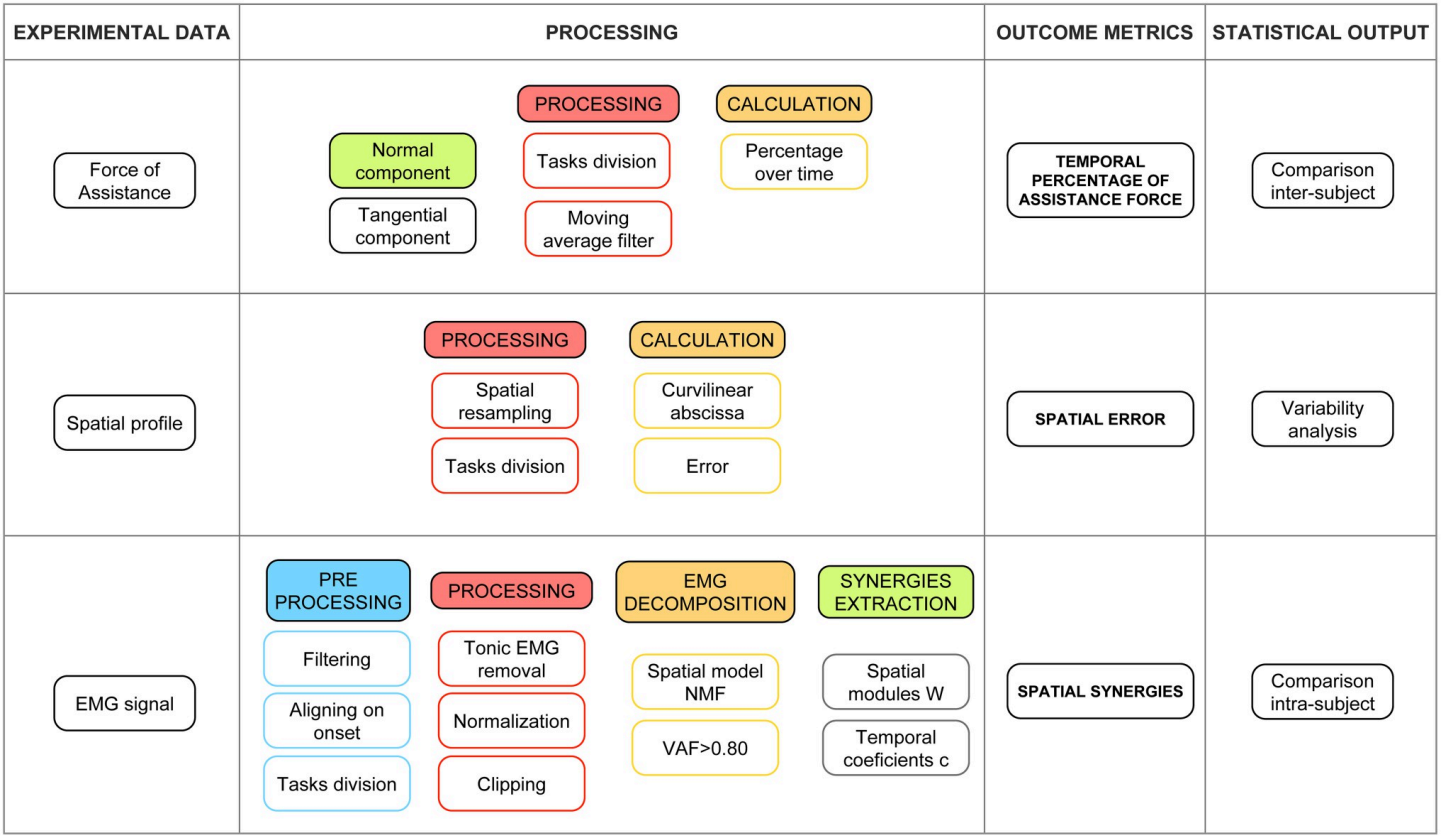
A detailed scheme of the 3 output variables is shown. 1) percentage of assistance force; 2) Spatial error in Free and Assisted modes with respect to the Reference Trajectories; 3) EMG data analysis and synergy extraction. EMG data were first aligned, filtered and segmented into sub-tasks. Then, EMG data underwent removal of tonic components, and each channel was normalized to the maximum EMG found in all trials for that channel. Lastly, negative phasic EMG data were clipped to 0. Then, synergies were extracted with the NMF algorithm; the analysis of human-robot interaction was conducted by comparing spatial synergies of the Free and Assisted modes, for each exercise separately, with an intra-subject analysis.

An overview of the data analysis workflow is portrayed in Fig 4.

#### Assistance

In the “Assisted” trials, we computed the amount of assistance provided with the robot as in Eq 1):

$$\%assistance_{task}\left(sub\right)=\frac{\sum_{1}^{N}Fon}{full\_duration\_of\_task}$$ (1)

where the numerator represents the summation of the time instants in which the force is greater than 0 (F_on_>0), while the denominator is the summation of all the time instants.*%assistance* was computed in all the path directions, including returns.

First, we investigated the assistance level, by testing the inter-direction *%assistance* variability within subjects for each of the considered modes. We stacked the error values for each trial repetition and direction of each subject and tiled them in a vector representing subject *i*. Then, data were averaged across repetitions. Data normality was tested for all the remaining distributions (Shapiro-Wilk test) and used the Kruskal-Wallis Test with directions as factor. Normality test was repeated for the Radial, the Curvilinear ¼ circle and the Curvilinear ½ circle exercises. Then, we tested the difference in assistance in Radial, Curvilinear ¼ circle and the Curvilinear ½ circle exercises with a Kruskal-Wallis Test with exercise as factor. For all the tests, the significance level was α = 0.05.

#### Kinematic error

The main metric for assessment of the performance in the “Free” and “Assisted” condition was the average root mean error found comparing the robotic end effector path in Reference mode with the Free and Assisted respectively.

The error on the path was defined as in Eq 2):

$$Error_{mode}\left(sub\right)=\sum_{task=1}^{n_{task}}\sqrt{{\left(x_{mode}\left(sub\right)-x_{i}\left(sub\right)\right)}^{2}+{\left(y_{mode}\left(sub\right)-y_{i}\left(sub\right)\right)}^{2}}$$ (2)

where *x* and *y* represent the coordinates of the movement in the plane of motion, mode represents the interaction mode (either Free or Assisted), *sub* is the considered subject and *i* the ideal trajectory for the considered direction.

The error was computed in all the path directions, including returns. We quantified the kinematic error comparing movement direction across subjects, by testing the inter-direction variability within subjects for each of the considered modes.

For each subject and each direction of motion, we stacked the error values for each trial repetition and tiled them in a vector representing subject *i*. This was repeated for all subjects. Data normality was tested for all distributions (Shapiro-Wilk test), and tested the inter-direction error difference with a Kruskal-Wallis Test with tasks as factor. Then, we tested the error difference in Free and Assisted modes with a Kruskal-Wallis Test with modes as factor. The test was repeated for both the Radial, the Curvilinear ¼ circle and the Curvilinear ½ circle exercises. For all the tests, the significance level was α = 0.05.

#### EMG signal and synergies extraction

The first step of the data analysis (for all conditions) consisted in pre-processing all the kinematics data with the upper-limb model implemented in the VICON Nexus System. Data was further processed and was performed with Matlab 2020, with custom-developed software. First of all, kinematic recordings were used to separate movement phases. Each acquisition was thus segmented in 16 movements. The segmentation was achieved by computing the velocity profile associated with the marker of the second metacarpal head of the dominant limb and used as a signal for detecting movement onsets and offsets.

Then, all the movements were aligned by considering the EMGs starting from 0.25 s before the task onset and 0.25 s after the task offset [-0.25 s; +0.25 s]. This procedure ensured capturing the complete EMG waveforms, which began before movement kinematic onset and ended up after having reached the target [29]. Differently from other study designs (e.g. locomotion), the nominal duration of the tasks was not fixed and could vary (more specifically, ½ circle tasks had double the nominal duration of radial and ¼ circle movements). Thus, a procedure for time-scaling was adopted before extracting spatial synergies to account for this. In the radial task, EMG signals were resampled with 25 samples before the start of the phase, 50 samples between the beginning and the end of each phase, and 25 samples after the end. In this way, every single radial phase was made of 100 samples. Very minor compression or dilatation of profiles was found as the nominal duration of each task was 0.5 s. In the curvilinear task, the signal was resampled with 25 samples before the beginning of the phase and 25 samples after the end; during the movement, 50 samples were used for ¼ circle case and 100 samples for the ½ circle. Very minor compression or dilatation of profiles was introduced as the nominal duration of each task was 0.5 s for ¼ circle (total 100 samples) and 1.0 s for ½ circle (total 150 samples). This approach ensured capturing the complete EMG waveforms, with very minor alteration of the EMG movement profiles, and with synchronization of the onset and offset of the movements. The data from 16 sEMG channels were band-pass filtered between 20–450 Hz (Butterworth filter, 7th order) to remove aliasing effects inside the sampling, rectified, low-pass filtered with a cut-off frequency of 6 Hz (Butterworth filter, 7th order) to extract the EMG envelope. Afterwards, the EMG envelopes were further analyzed to extract the phasic component of the EMG, removing the postural (tonic) EMG activity from the original signal [30], following the approach used in previous works [29]. Following this approach, slightly negative EMGs could be obtained in some cases. In order to perform the non-negative matrix factorization (NMF), negative phasic EMG values were set to zero before synergy extraction. Lastly, a normalization procedure was performed in order to allow intra-subject comparisons.

Since our study design has a distinctive feature of mapping muscle patterns over a variety of movement directions of the upper-limb horizontal workspace, we aimed to refer the variability captured in the EMG recordings to the whole dataset of each participant (both exercises and both assistance conditions). Since Maximum Voluntary Contraction (MVC) is very hardly measured on some muscles, the normalization of the data was performed on the maximum value achieved for each muscle in the complete dataset, using a metric that includes all movements (“Global Normalization”, already employed in previous studies [28, 29, 31]).

For each subject, the aligned, filtered, de-tonified, normalized and zero-clipped EMG envelopes were arranged to generate the pooled matrix data to be given as input to the synergy extraction algorithm: for each subject, and for each of the two experimental modalities (Free and Assisted) and in all trajectories (Radial and Curvilinear), all the movements for each trial and from each condition were grouped together for each specific experimental condition.

Summarizing, each EMG pooled matrix had dimensionality [(n_samples_)*n_tasks_] x [n_muscles_], stacking together data from the 10 repetitions of each task [32]. In detail, n_tasks_ = 16 (radial or curvilinear movements), n_muscles_ = 16 (EMG channels); in the Radial case n_samples_ = 100, while in the Curvilinear case, n_samples_ = 100 (¼ circle) and n_samples_ = 150 (½ circle). In the curvilinear case, the number of samples was adapted to the length of the movement to provide a uniform spatial sampling of the trajectory. Then, spatial synergies were extracted separately for the three tasks using the NMF algorithm [33]. The NMF decomposes the EMG data matrix into the product of two matrices, the first one representing time-invariant synergies (w_i_), and the second one representing time-varying activation coefficients for each synergy (c_i_), as in Eq 3):

$$EMG\left(t\right)=\sum_{i=1}^{N}c_{i}\left(t\right)w_{i}$$ (3)

where, for each of the recorded muscles, *EMG(t)* represents the EMG data at time *t* and *N* is the total number of extracted synergies.

Thus, for each decomposition, each spatial synergy was coupled with a set *n* of coefficients (dimensionality *n* equal to the total number of time samples of all included movements); each set had dimensionality *N* equal to the number of extracted synergies.

The order of the factorization *r* given as input to the NMF algorithm was chosen increasingly from 1 to 16 (the maximum number of muscles that characterizes the maximum dimensionality of the examined control problem). For each *r*, the NMF algorithm was applied 100 times in order to avoid local minima and the repetition accounting for the highest fraction of total variation of the signal explained by the synergy reconstruction was chosen as the representative of order *r*.

The number of synergies *N* was then chosen as the minimum *r* explaining at least 80% of the data variation, quantified by a reconstruction R^2^ defined as 1 –SSE/SST where SSE is the sum of the squared residuals, SST is the sum of the squared differences with the mean EMG vector [29].

**Fig 5:**
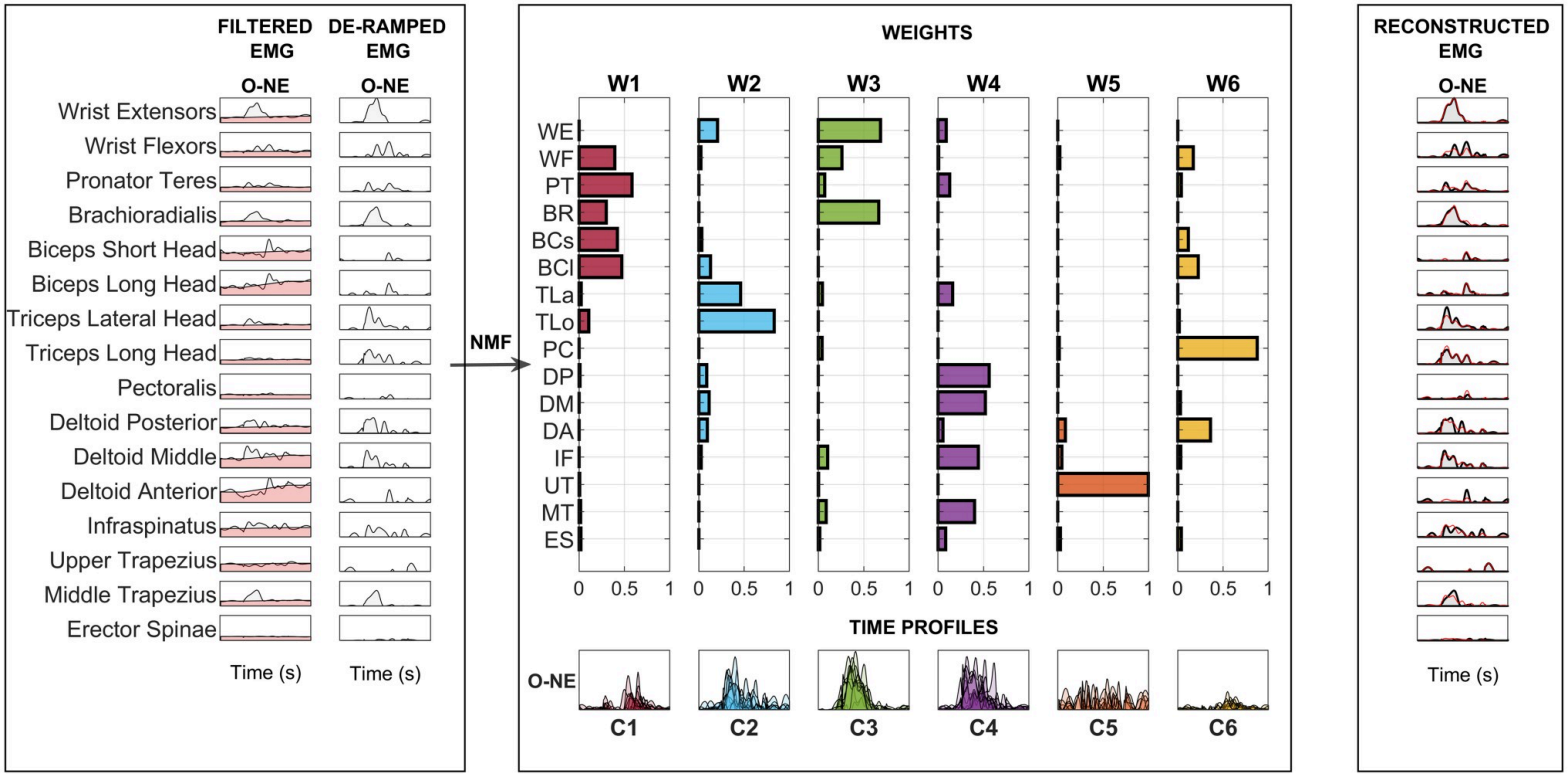
EMG elaboration and spatial synergies extraction. In the left panel, the aligned and filtered EMG envelopes are reported in light gray for a representative subject, condition and task (O-NE). In light red, the ramp model for tonic activity is portrayed. The phasic EMG activity is achieved by subtracting the tonic activity from the original envelopes. The normalization and zero-clipping procedures have been applied in order to remove the negative phasic components (center panel) and apply the standard NMF method. In the center panel, we showed the extracted spatial synergies and the temporal coefficients in the same representative task. The C coefficients are here shown for all the 10 repetitions, overlapped one with the other. In the right panel, in red, the reconstructed EMG envelopes are shown for a representative subject and condition respectively. In light gray, the original EMG envelopes are reported.

In Fig 5a–5c the procedure for synergy extraction is shown in a representative task.

Filtered and aligned waveforms include the contributions from phasic and tonic EMG activity. Regarding EMG data processing, we removed the contribution of the tonic activity according to a ramp model [29]. Normalization and zero-clipping procedures have been applied in order to remove the negative phasic components and apply the standard NMF method to extract muscle synergies [28, 29].

Then, synergies (time-invariant spatial loads W) and temporal coefficients (C) were extracted.

For each subject, the extracted spatial synergies in Free and Assisted conditions were matched by similarity so that each synergy was coupled with the more similar one found in the other dataset [34, 35]. Then, each couple of matched synergies was assigned a similarity score (cosine product between the vectors containing the synergy loads for coupled synergies). This analysis was repeated for both the Radial and Curvilinear (¼ circle and ½ circle) exercises. Data normality was tested for all distributions (Shapiro-Wilk test). To measure the effects of assistance on muscle synergies and detect whether it was comparable on all our enrolled subjects, we run the Kruskal-Wallis Test for each Free-Assisted similarity level having subjects as factor (level of significance α = 0.05). This was repeated for both Radial and Curvilinear (¼ circle and ½ circle) tasks. We also tested the mean similarity of the paired synergies when comparing the Assisted and Free mode with Radial and Curvilinear (¼ circle and ½ circle) exercises as factors.

Since high or very high synergy similarity was found between the Free and Assisted modes, we also provided an analysis of temporal coefficients associated with synergies. Each extracted synergy was modulated with temporal coefficients for each of the directions of motion; thus, for each paired spatial synergy, we computed the Pearson correlation coefficient for the temporal coefficients in each of the directions, comparing the Free and Assisted modes. Then, for each pair of matched spatial synergies, we averaged the Pearson correlation of temporal coefficients across directions to achieve a synthetic score for temporal coefficient similarity to be coupled to each spatial synergy. This score was computed only on spatial synergies having similarity = 0.60 or higher, since it is not meaningful to compare temporal coefficients for synergies that are not reasonably high spatially matched. Then, data normality was tested for all distributions of temporal coefficient similarity (Shapiro-Wilk test). To measure the effects of assistance on temporal matching and detect whether it was comparable on all our enrolled subjects, we run the Kruskal-Wallis Test for each Free-Assisted temporal similarity score, with subjects as factor (level of significance α = 0.05). This was repeated for both Radial and Curvilinear (¼ circle and ½ circle) tasks. We also tested the mean similarity of the temporal coefficients matching score when comparing the Assisted and Free mode with Radial and Curvilinear (¼ circle and ½ circle) with the Kruskal-Wallis Test, using the exercises as factors.

#### Summary of statistics

We hereby summarize the statistical analysis for this study. Throughout the analyses, we analyzed 3 factors: Directions (comparing e.g. O-NE, O-E,… in the Radial case and all the different ¼ circle and ½ circle in the Curvilinear one); Modes (comparing Free and Assisted mode); Exercises (comparing Radial and Curvilinear (¼ circle and ½ circle) exercises). In all conditions, normality of the data was tested with the Shapiro-Wilk test, Matlab *swft function*. Since many distributions were not normal, the non-parametric Kruskal-Wallis Test was used.

## Results

Participants age was 33±9 years, 2F, 8M. The study was approved by the Politecnico di Milano Ethical Committee. All subjects gave consent to participate and signed a written informed consent before the experiment, which was conducted in accordance with the Declaration of Helsinki.

### Kinematic error

**Fig 6:**
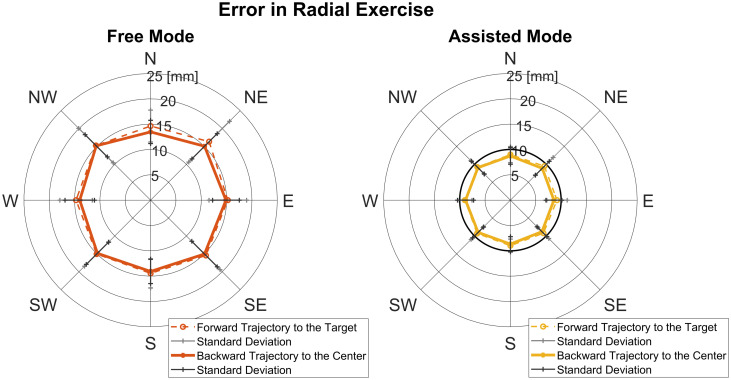
Radial kinematic error. Mean inter-subject error and deviation in the Radial mode displayed for trajectories towards the targets and back to the center. For the Assisted mode, we also displayed the radius of the assistance tunnel in black.

In Fig 6, the kinematic error is reported for the radial tasks.

In the Radial tasks, we found that, across directions, there was no statistical difference in the Free (p = 0.99) and in the Assisted (p = 0.99) modes.

**Fig 7:**
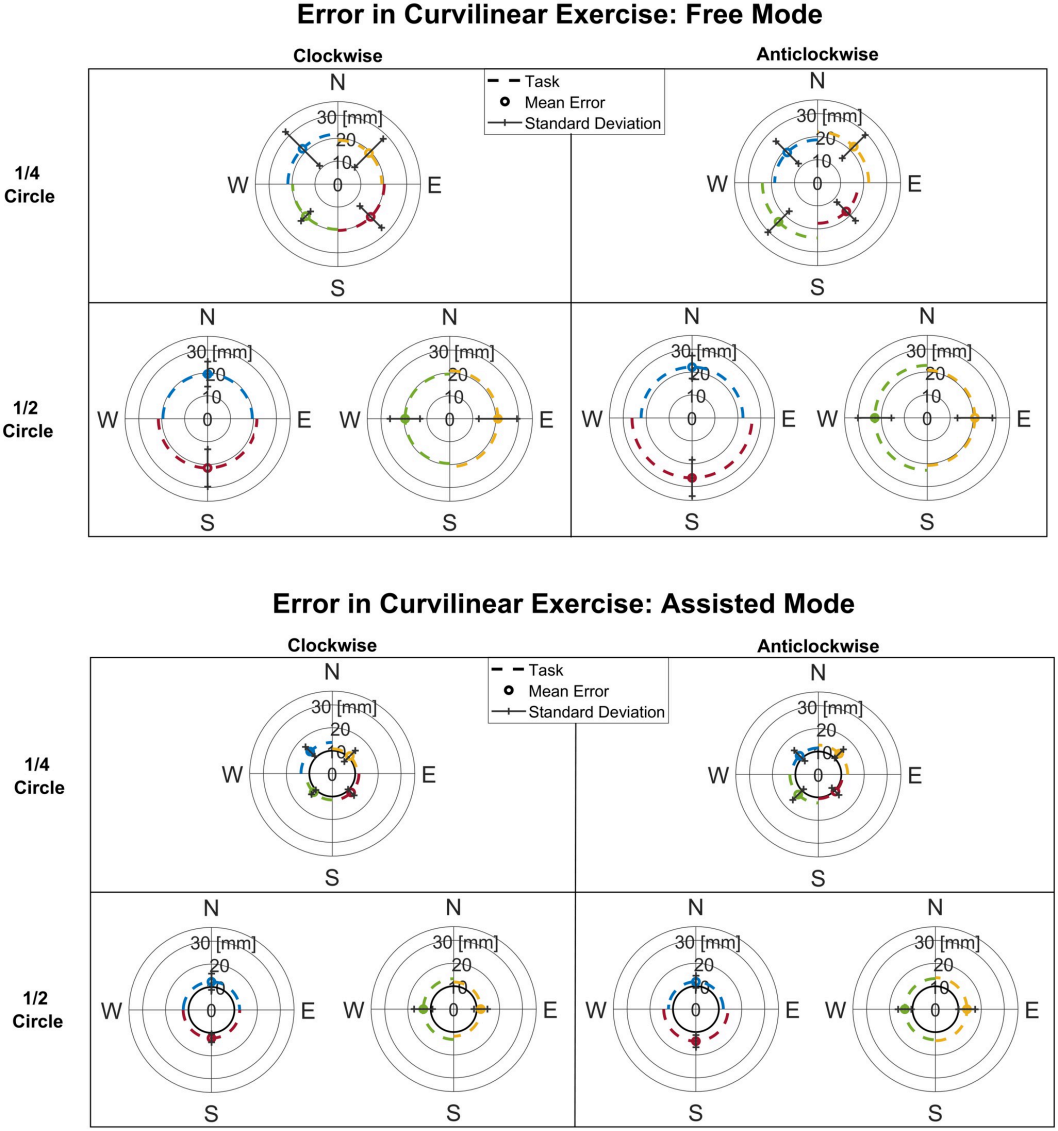
Curvilinear kinematic error. Mean inter-subject error and deviation in the curvilinear tasks in Free mode (upper panel) and in Assisted mode (lower panel) displayed for ¼ circle and ½ circle and for direction of execution (clockwise and anticlockwise). For the Assisted mode, the radius of the assistance tunnel is displayed in black.

In Fig 7 (upper panel and lower panel), the kinematic error is reported for the curvilinear tasks. In the Curvilinear exercise, we found that, across directions, there were no statistical difference in the Free ½ circle (p = 0.13), in the Free ¼ circle (p = 0.19), in the Assisted ½ circle (p = 0.17) and Assisted ¼ circle (p = 0.19) mode.

**Fig 8:**
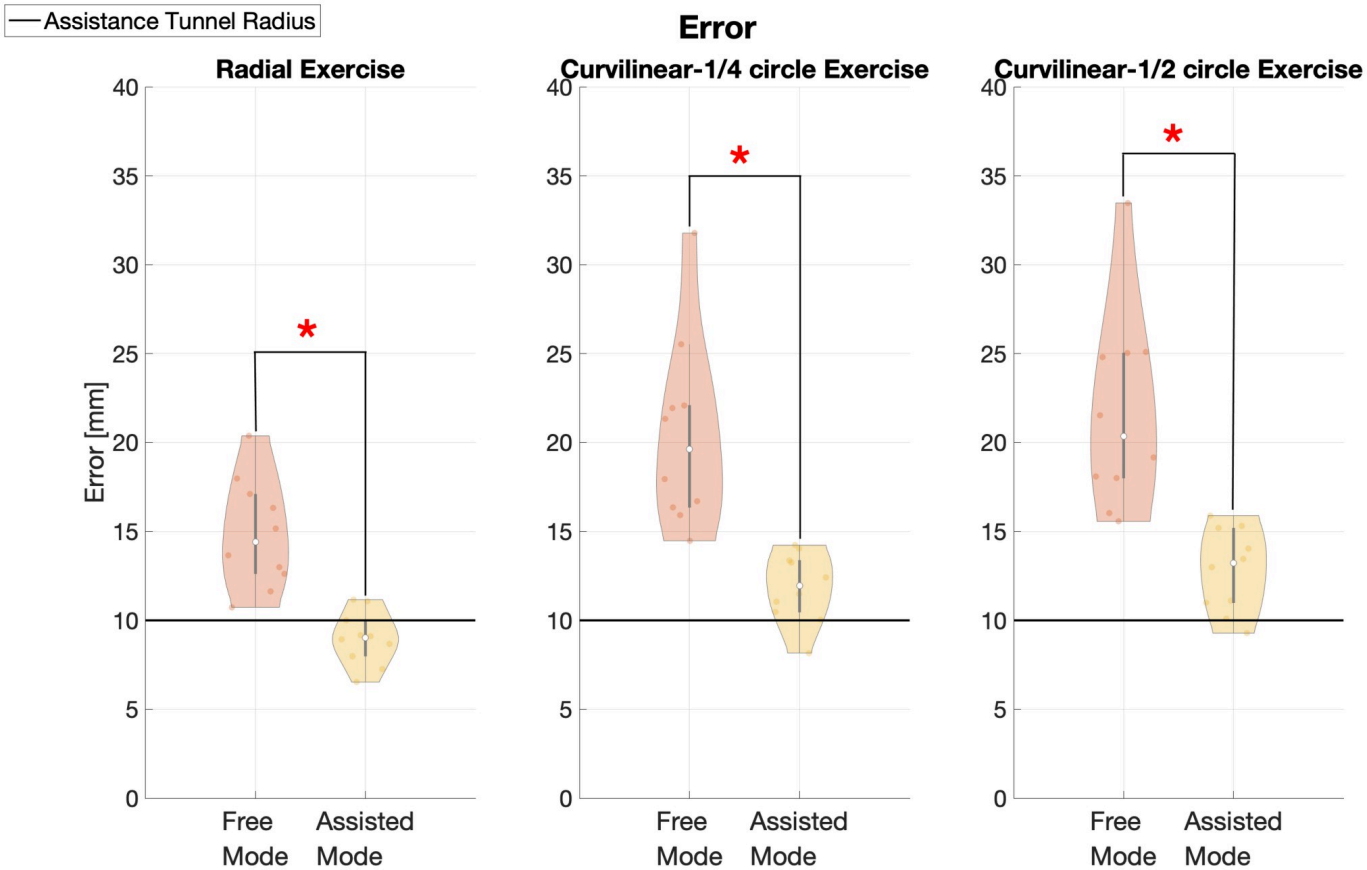
Summary of kinematics errors in the three modes. left panel: Radial free and Radial assisted; middle panel: Curvilinear free ¼ circle and Curvilinear assisted ¼ circle and right panel: Curvilinear free ½ circle and Curvilinear assisted ½ circle. The mean kinematic error after repetition averaging and across directions is shown for each subject. We displayed the radius of the assistance tunnel in black.

In Fig 8, the kinematic error is reported comparing Free and Assisted conditions in each exercise. The kinematic error in the Free mode were significantly higher than in the Assisted one in Radial (Mean error Free = 14.85±3.07 mm, mean error Assisted = 8.99±1.50 mm, p = 0.0003*); in Curvilinear ¼ circle (Mean error Free = 20.40±5.30 mm, mean error Assisted = 11.85±1.96 mm, p = 0.0002*) and in Curvilinear ½ circle (Mean error Free = 21.68±5.52 mm, mean error Assisted = 12.83±2.34 mm, p = 0.0002*).

These results underline the effect of the assistance during exercises, reducing the kinematic error in both modes. Moreover, as expected assistance led the subjects to an average error lower than the size of the tunnel in the Assisted mode.

In detail, it was noted that the error in the Free mode was higher in respect to the Assisted mode. The mean error of Curvilinear (¼ circle, ½ circle) exercises (challenging condition) resulted higher than the Radial.

### Assistance level

**Fig 9:**
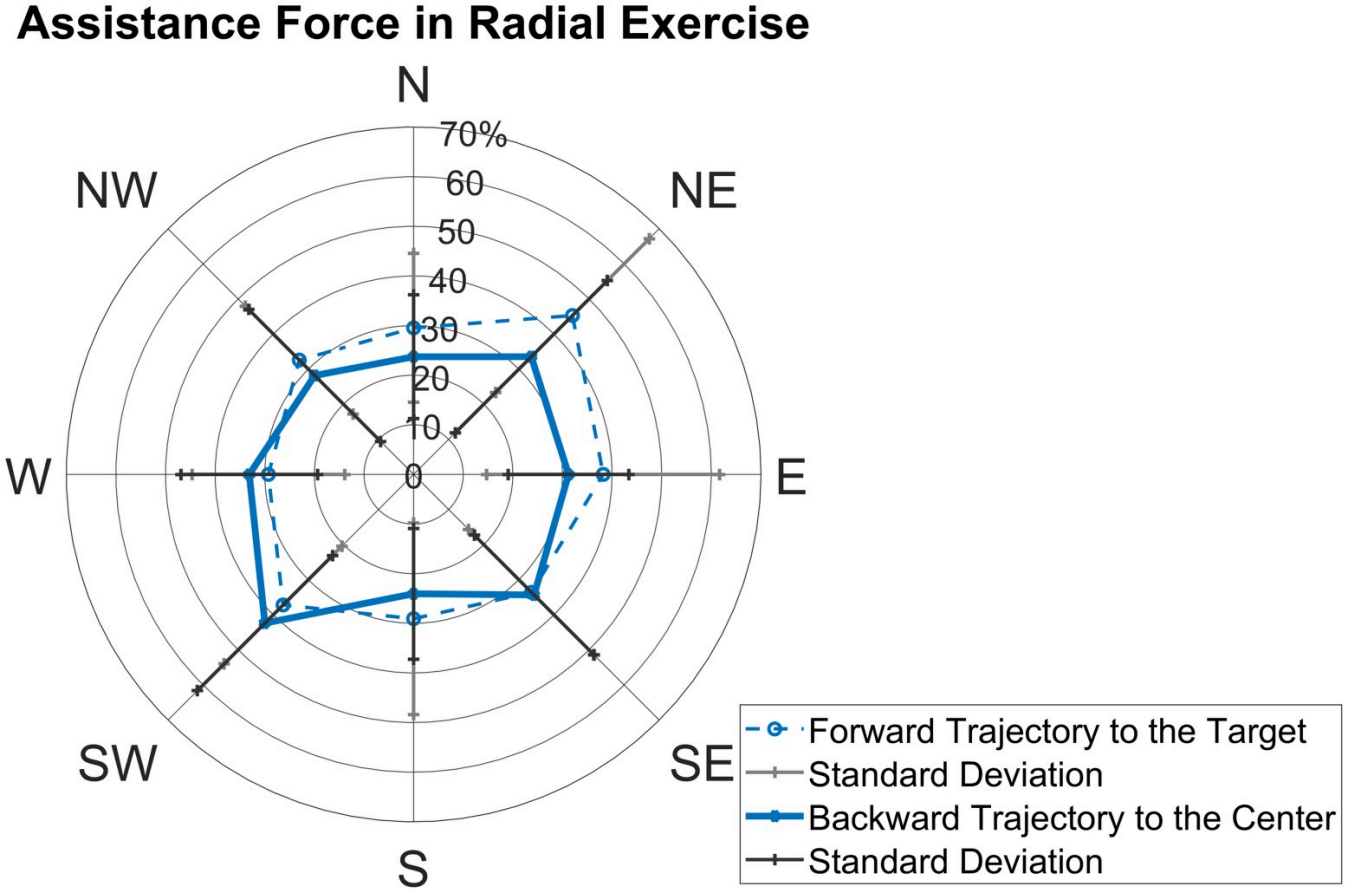
Radial assistance level. Mean inter-subject assistance and deviation in the Radial task, displayed for trajectories towards the targets and back to the center.

The level of assistance is reported for the Radial and Curvilinear exercises. In Fig 9, the assistance level is reported for the Radial exercise. We found that there was no statistical difference among directions (p = 0.36).

**Fig 10:**
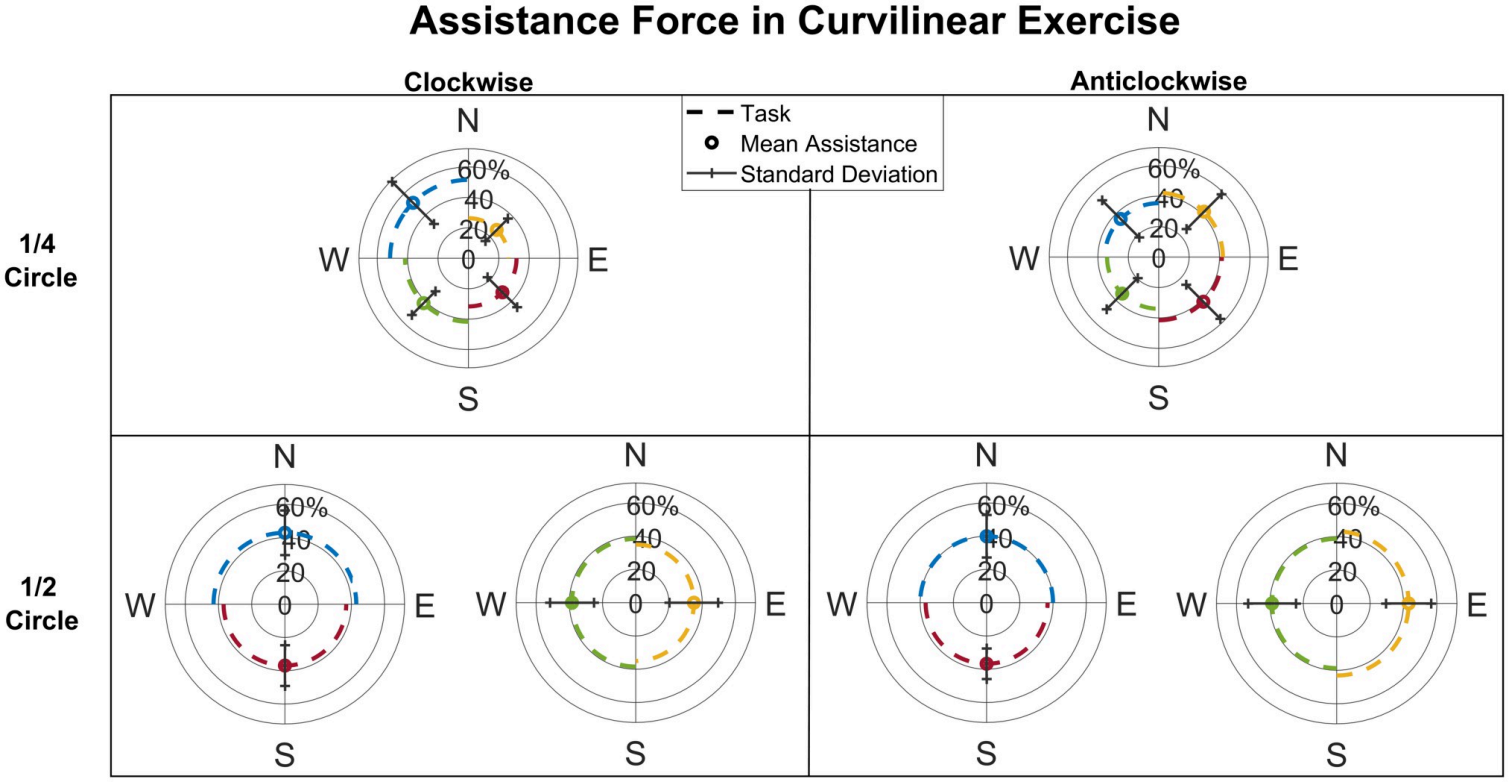
Curvilinear assistance level. Mean inter-subject assistance and deviation in the Curvilinear mode, displayed for ¼ circle and ½ circle and for direction of execution (clockwise and anticlockwise).

In Fig 10, the assistance level is reported for the Curvilinear tasks. We found that there was no statistical difference across directions in the Curvilinear ½ circle (p = 0.33) and in the Curvilinear ¼ circle (p = 0.01*). The multiple comparison test between all directions in the Curvilinear ¼ circle revealed that there is an assistance difference only between the trajectory of the ¼ circle W-N and that N-E in a clockwise direction (p = 0.007*).

**Fig 11:**
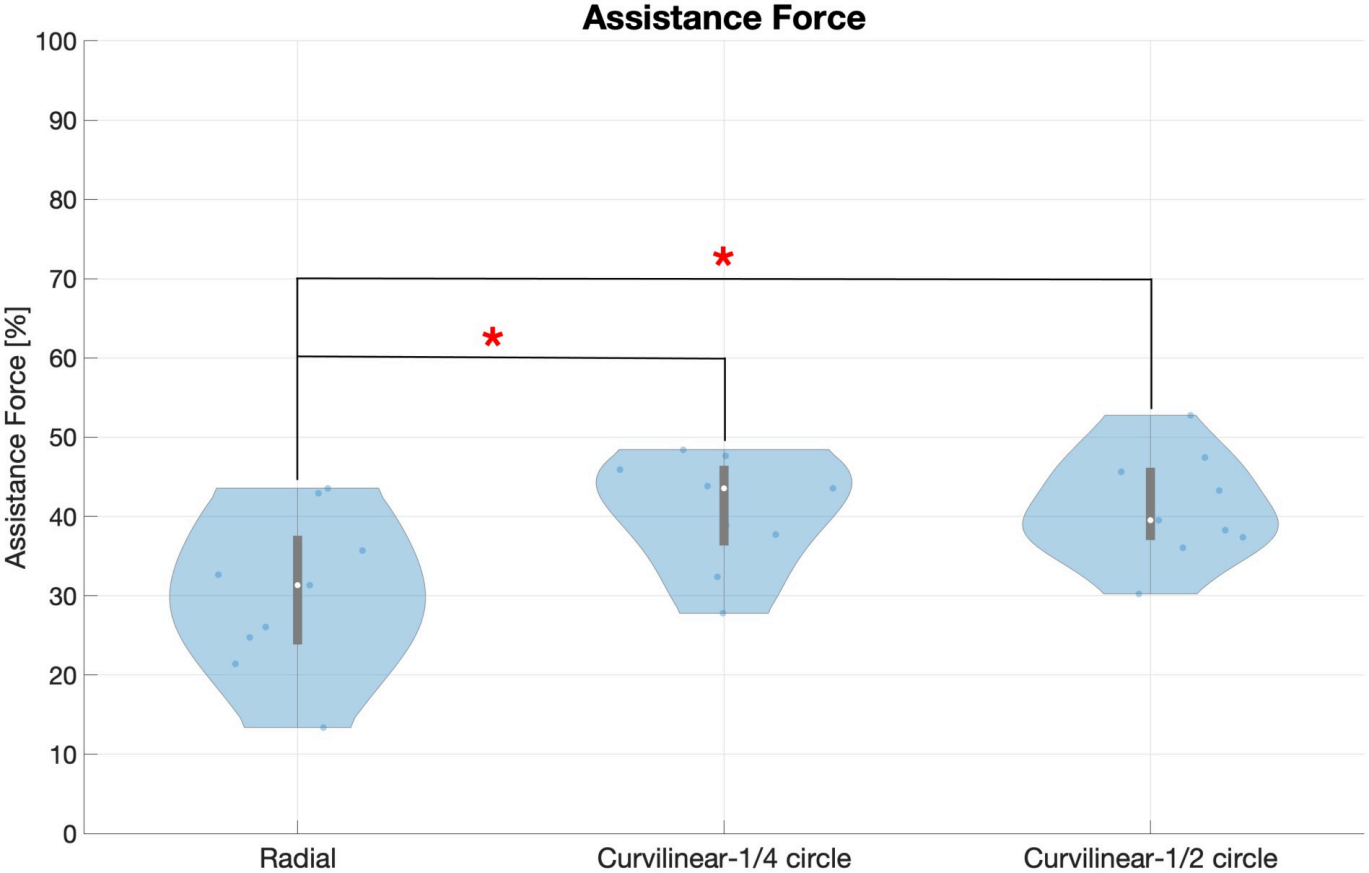
Comparison of assistance level in Radial and Curvilinear movements. The assistance level in Curvilinear ½ and Curvilinear ¼ exercises is higher than in Radial. The mean assistance level after repetition averaging and across directions is shown for each subject.

In Fig 11, the level of assistance is reported comparing the Radial and Curvilinear (¼ and ½ circle) exercises. We found that the assistance in the Curvilinear exercises is in general higher than in the Radial: in detail, the average of the temporal percentages of assistance is respectively 30±9% in the Radial exercise; 41±7% in the Curvilinear ¼ circle and 41±6% in Curvilinear ½ circle (p = 0.021*). Multiple comparison test between these three conditions shows evidence for a statistical difference between Radial—Curvilinear ¼ (p = 0.03*).

We conclude that assistance is higher in the Curvilinear exercises, which represent more challenging tasks compared to the reaching movements of the Radial exercise.

### Synergy analysis

#### Number of extracted synergies

In Radial movements, the mean number of extracted synergies was 6±1 in Free mode and 6±1 in Assisted mode (p = 0.84, no statistical difference); in Curvilinear movements, the mean number of synergies was 6±1 in Free mode and 6±1 in Assisted mode (p = 0.82, no statistical difference).

These results show that the number of motor modules extracted is in general not modified with exercise and mode. We also found that there was no statistical difference between the number of extracted synergies of the two modes in the Radial and Curvilinear when considered together (p = 0.98). Following these results, we decided to set the number of synergies to 6 for all subjects and conditions in order to be able to always match synergy composition between modalities.

#### Extracted synergies matching

**Fig 12:**
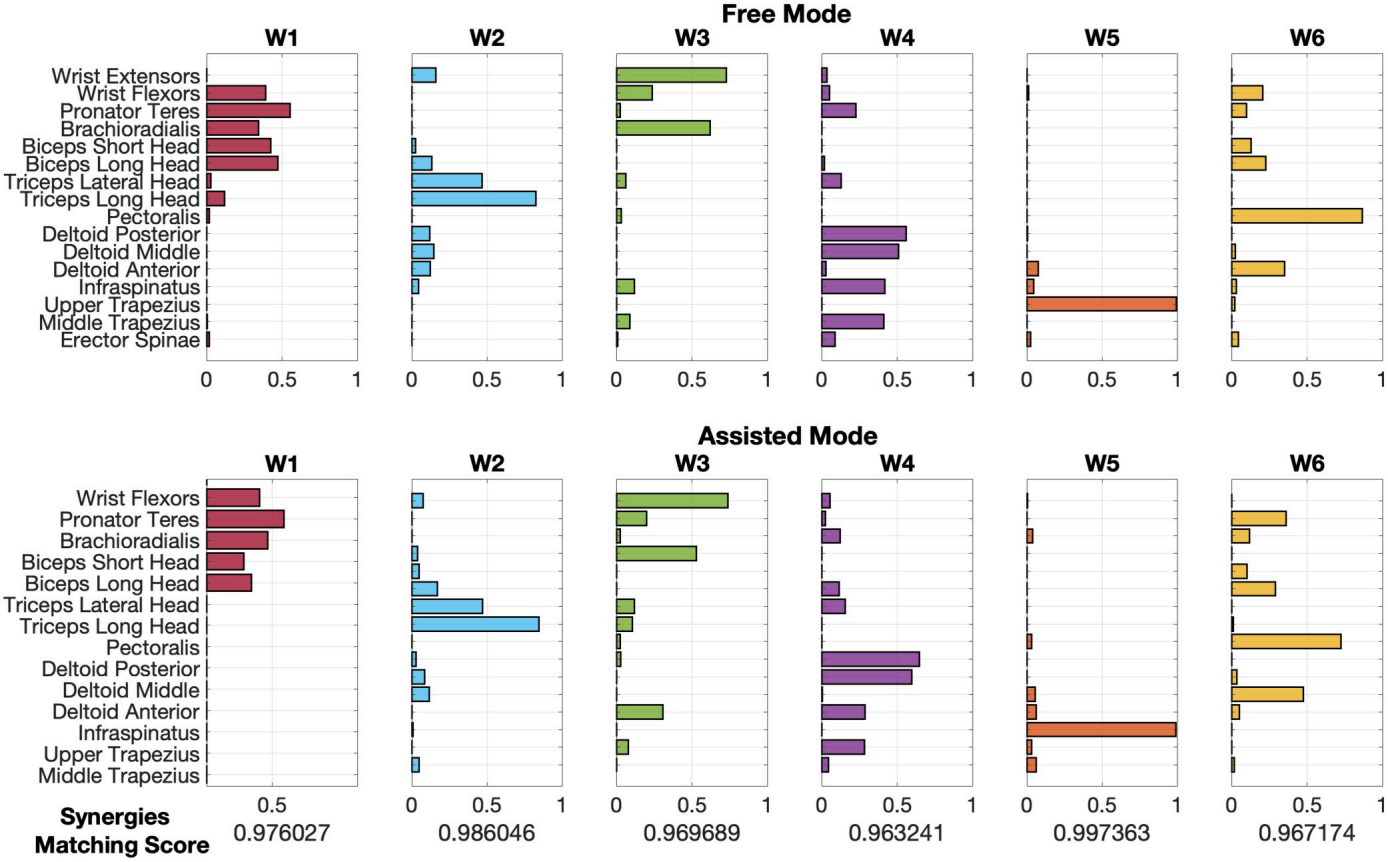
A typical example of paired synergies coupled by similarity following the extraction in the Radial Free and Assisted for the subject 1. We found that the matching score for muscle synergies (W) in the two modes is high: in our trial, assistance influenced task performance, but only a slight modification of muscle synergies was found.

In Fig 12, we report an example of the matching between the paired synergies of the Free and Assisted datasets.

**Fig 13:**
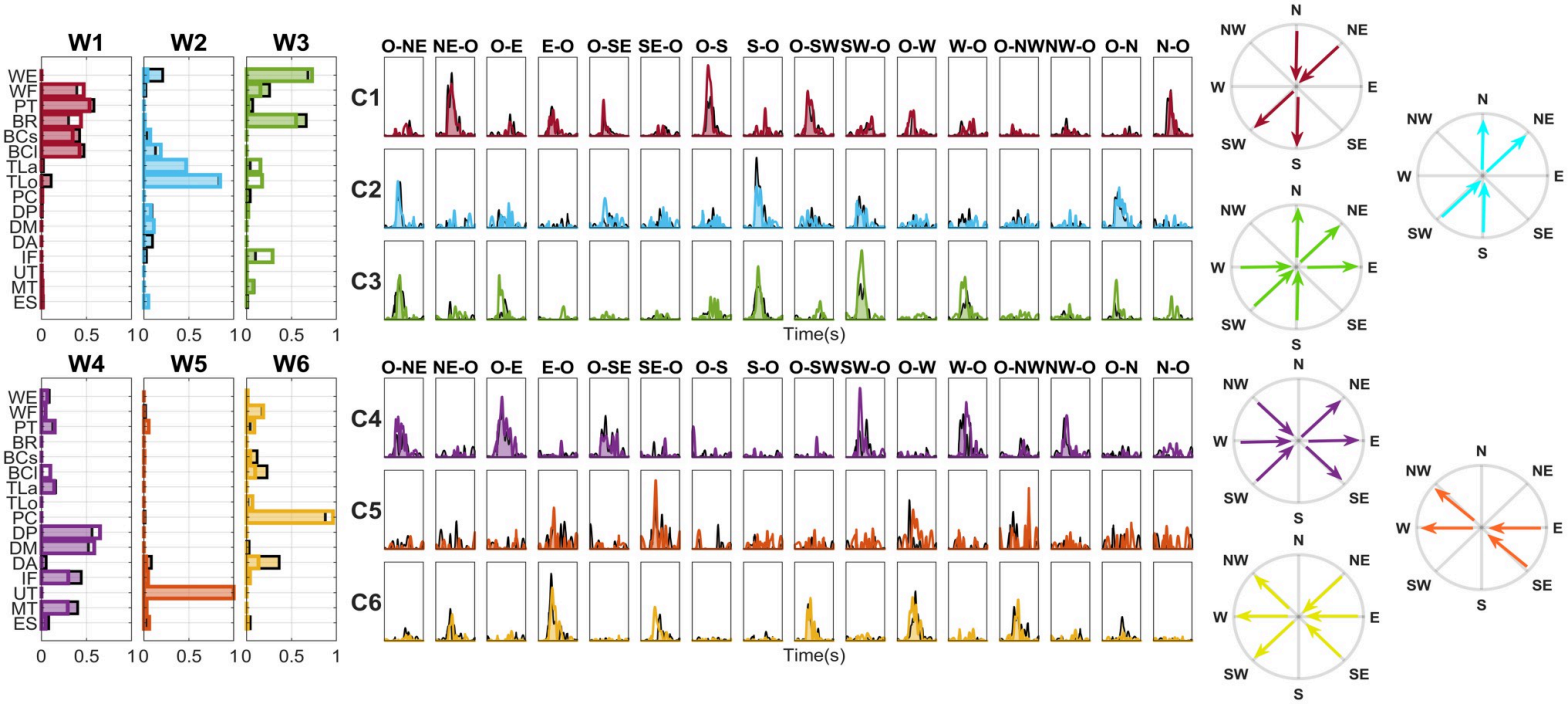
For a typical subject, comparison between spatial synergies, temporal coefficients, and directional tuning. On the left, the spatial synergies of the two modes are represented overlapped (Free data have a black edge and a colored light area; Assisted data have a marked colored edge and an empty area). In the center of the Figure, the temporal coefficients of the MS are portrayed, with the same colorcode used for spatial synergies. On the right, the directional tuning of spatial synergies is portrayed.

In Fig 13 we extend the results found on a typical subject (Subject 1, Radial exercise), also showing temporal coefficients *c* and the main directional tuning related to each synergy.

For example, analyzing the muscle activations and the temporal coefficients, a flexor synergy at elbow/forearm level is found in W1 (red synergy loads); an extensor pattern at elbow level is found in W2 (light synergy loads); W3 includes extension at wrist level (green synergy loads); another extensor pattern at the shoulder level is in W4 (purple synergy loads); W5 shows a trapezius activation, probably to keep the arm elevated (orange synergy loads); W6 rotates the shoulder internally towards the medial hemispace (yellow synergy loads).

Comparing qualitatively Free and Assisted data, we show that, for this subject, the directional tuning of synergies is preserved. In further studies, these aspects will be investigated in more detail.

#### Effects of assistance on muscle synergies

After coupling muscle synergies extracted in the Free and Assisted modes (for the Radial, Curvilinear ¼ circle and Curvilinear ½ circle tasks), we quantified the mean distributions of synergy matching scores for each subject.

**Fig 14:**
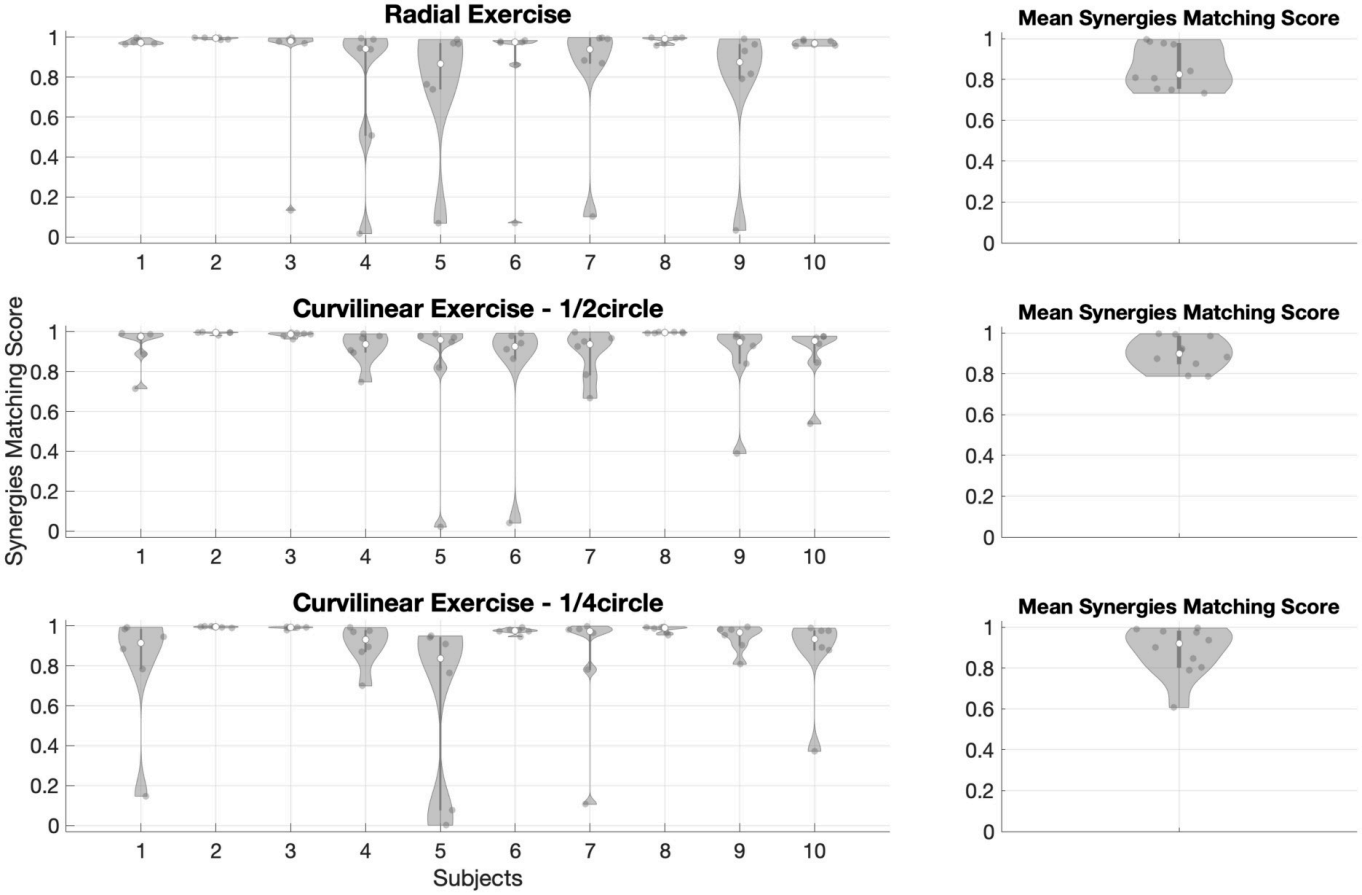
Distribution of mean synergy matching scores (Free vs Assisted). It is portrayed in the Radial exercise (first row), Curvilinear ½ circle exercise (second row), Curvilinear ¼ circle exercise (third row). Our results show that the synergy matching is generally high. In the Radial exercise, statistical tests showed that some subjects’ synergy matching is different from others (subject 5 and subject 9 matchings are lower than subject 2’s); in the Curvilinear ¼ circle exercise, statistical tests showed that some subjects’ synergy matching is different from others (subject 2 and subject 3 matchings are lower than subject 5’s); in the Curvilinear ½ circle exercise, statistical tests showed that some subjects’ synergy matching is different from others (subject 9 and subject 10 matchings are lower than subject 8’s). The mean Synergies Matching Score for all subjects is reported for both exercises.

The values for synergy matching for Radial and Curvilinear (¼ circle and ½ circle) tasks are shown for all subjects in Fig 14.

In the Radial exercise, the statistical test showed statistically significant differences between subjects (p = 0.0194*). The multiple comparison test between all subjects revealed that subjects with this different similarity are found when comparing the following pairs: subject 2-subject 5 (p = 0.04*) and subject 2-subject 9 (p = 0.03*); moreover, subject 2 and subjects 5 and 9 were not different when compared to all other subjects.

The statistical test about Synergy matching scores for Curvilinear ¼ circle tasks detected significant differences between subjects (p = 0.0009*). Post-hoc tests revealed that this difference is found when comparing the following pairs: subject 5-subject 2 (p = 0.0016*) and subject 5-subject 3 (p = 0.0148*); moreover, subject 5 and subjects 2 and 3 were not different when compared to all other subjects.

The statistical test about Synergy matching scores for Curvilinear ½ circle tasks detected significant differences between subjects (p = 0.0007*). Post-hoc tests revealed that this difference is found when comparing the following pairs: subject 8-subject 9 (p = 0.048*) and subject 8-subject 10 (p = 0.037*); moreover, subject 8 and subjects 9 and 10 were not different when compared to all other subjects.

Considering the mean value of synergy matching score for all the subjects to compare the Radial (Mean synergies matching score between all subjects = 0.86±0.18), Curvilinear ¼ circle (Mean synergy matching score = 0.88±0.16) and Curvilinear ½ circle (Mean synergy matching score = 0.89±0.13) exercises, we found that there was no statistical difference (p = 0.642).

In general, synergy matching scores were very high: the effect of assistance on the extracted muscle synergies in Curvilinear (¼ circle, ½ circle) and Radial movements is in general limited, even if not completely uniform.

#### Effects of assistance on temporal coefficients

The results on the comparison of paired temporal coefficients are presented in this paragraph.

**Fig 15:**
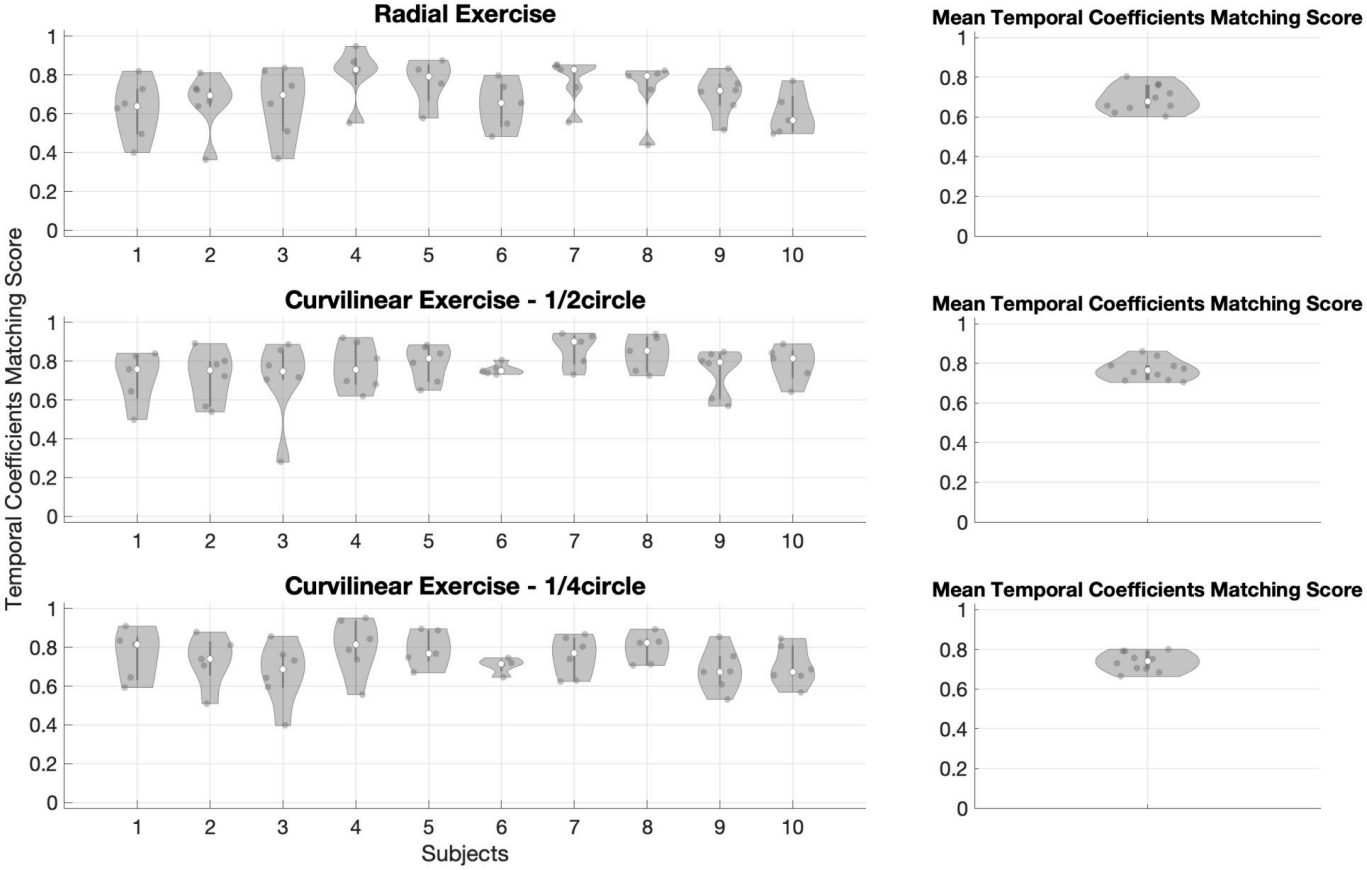
Distribution of temporal coefficients matching scores (Free vs Assisted). It is portrayed in the Radial exercise (first row), and Curvilinear ½ circle exercise (second row), Curvilinear ¼ circle exercise (third row). Our results show that the temporal coefficient matching is generally quite high. In the Radial exercise, in the Curvilinear ¼ circle exercise, and in the Curvilinear ½ circle exercise, no statistically significant differences between subjects were shown. The mean Synergies Matching Score for all subjects is reported for both exercises.

The matched score for temporal coefficients (Free vs Assisted) associated with spatial synergies for Radial and Curvilinear (¼ circle and ½ circle) tasks are shown for all subjects in Fig 15.

In the Radial exercise, in the Curvilinear ¼ circle tasks and in the Curvilinear ½ circle tasks, the statistical test did not show statistically significant differences between subjects (p = 0.181, p = 0.551, and p = 0.540, respectively).

Considering the mean value of temporal coefficient matching score for all the subjects to compare the Radial (Mean temporal coefficients matching score = 0.69±0.07), Curvilinear ¼ circle (Mean temporal coefficients matching score = 0.74±0.05) and Curvilinear ½ circle (Mean temporal coefficients matching score = 0.77±0.05) exercises, we found that there was no statistical difference (p = 0.063). In general, temporal coefficient matching scores were quite high: the effect of assistance on the extracted muscle synergies in Curvilinear (¼ circle and ½ circle) and Radial movements is in general limited, even if not completely uniform.

## Discussion

### Summary of the main results

In this study, we examined the effect of the force-tunnel assistance paradigm on healthy people in standard and challenging upper-limb training conditions as a first step for a deeper comprehension of mechanisms of human-robot interaction. Our results showed that assistance and error are higher in challenging conditions, but muscle synergies are only slightly affected both in standard and challenging exercises. Temporal coefficients are moderately affected but preserve a good correlation when comparing the Free and Assisted modalities.

### Effects of robotic assistance on healthy people in upper-limb training

In our study, we found that the paradigm for assistance based on force tunnel allows to improve task performance in standard Radial exercises and in challenging Curvilinear tasks in healthy subjects. At the same time, this result is achieved with more assistance intervention in Curvilinear tasks in respect to Radial. This result was in line with the initial hypothesis that challenging conditions would require more robot assistance even in healthy subjects.

In this study, we wanted to link the intervention of the robot assistance to the motor control strategies adopted by the subject, comparing muscle synergies between Free and Assisted modalities. A reduced number of preliminary works have investigated how robot devices influence control at synergy level; in fact, the effect of robotic rehabilitation devices on muscle synergies has rarely been reported in the scientific literature, and mostly on healthy people. Most of the studies focus on the acute effect of adaptation to devices, showing how synergies are affected during human-robot interaction in respect to free motion. Coscia et al., [36] reported that spatial muscle synergies undergo only slight variation when healthy subjects are assisted with a device for weight support, while temporal coefficients are modified (reduced in magnitude). Similarly, Pirondini et al. [37] used muscle synergies as outcome measure to show that muscle activity of healthy volunteers during assisted movements was reduced with respect to the movements performed actively.

Even if a planar set-up is employed in this study, our results are in accordance with these previous findings, showing that assistance allows improving task performance (i.e., reducing errors in respect to ideal trajectories), but only alters the spatial structure of muscle synergies in a limited way. We conclude that in our planar scenario, including standard and challenging motor gestures in a wide variety of directions, healthy people synergies are often invariant or slightly modified with the intervention of robot assistance. This result is even more relevant because it extends previous findings to purely-phasic motion-related synergies, after tonic EMG components removal; in fact, in all the enrolled subjects, the phasic (movement-related) synergies are very well matched between the Free and Assisted modes. These findings may support the employment of robotic devices as training operators. In fact, while robots are needed to support correct motion to assure completion of tasks and avoid frustration, their use has so far focused on principles of minimal assistance [38], i.e., the robot should intervene only when necessary. It follows that device transparency is a desirable feature [39]. The framework of assist-as-needed paradigms, where robot intervention comes into play only when subject error overpasses a certain threshold (which can be defined in multiple ways) [40] was in fact conceived not to alter the process of motor learning that passes through trial-and-error learning process which should be experimented by the user [41]. However, the intervention of the robot could in principle alter the motor control strategy adopted by the subject, acting as an external perturbation, despite achieving a reduced error. Verifying that robotic assistance is not disrupting the muscular synergies adopted by healthy subjects was the main rationale for the present experimental study as it was experimentally found. When we address the synergies extracted on healthy people, we observe outcomes proving that with assistance, performances did improve, and this was achieved with a slight alteration of the synergic modules employed for limb control. This finding is also consistent with the concept of invariant synergies within a class of similar movements and has an important impact in the design of rehabilitation robots. Temporal coefficients had a medium-high correlation. Their matching was not as high as spatial synergies; this was expected as synergies are not perfectly matched (similarity < 1) and thus, it is reasonable to achieve lower values for temporal coefficients. However, temporal coefficient similarity is high enough to clearly identify an underlying solid control structure, especially considering the variety of exercises performed, including challenging motion conditions. We also mention that the choice of processing (e.g., fixing a priori the number of extracted synergies) may partially impact on the results. In non-reported partial results, even higher synergy matching was found when non-imposing a priori the number for extracted synergies; however, since non-matched synergies were not considered in the computation, the similarity achieved was probably slightly inflated with this approach (not presented).

Previous works also examined the effect of robotic operators to assist lower-limb motion in pilot studies on healthy people. In Moreno et al. [42], muscle synergies were extracted from Lokomat assisted walking when varying interaction modalities (velocity and ground forces magnitude). Authors concluded that in many of the proposed conditions, muscle synergies and their temporal coefficients are only slightly altered in respect to control walking. Our paper thus extends previous results achieved in a very controlled and repeatable movement (locomotion) to a variety of upper-limb movements. Of course, our results should be considered valid for the repertoire of gestures and assistance paradigms used in this work and cannot be generalized to all upper-limb assisted robotic training set-ups.

Nevertheless, the understanding on how to translate these findings to clinical practice is not obvious. Only a few studies report the use of synergies as outcome measures for robotic therapy on patients. However, none of these concentrated on the effects of human-robot interaction, but rather used synergies to measure the effect of the therapy. Tropea and colleagues [21] investigated the effect of planar training and concluded that the modification of synergies on patients followed a specific pattern for each subject. A recent study [20] showed that training provided with a planar robot can successfully modify abnormal muscle synergies to resemble the healthy ones in the proximal district. However, there have been negative effects in the control of the distal district which could be improved by adding other forearm-specific rehabilitation or distally targeted robot-assisted therapy. Scano et al. [22] reported that the effect of robotic 3D training induced slight alteration of spatial synergies in respect to free movement in post-stroke patients.

While very few studies are available to evaluate human-robot interaction with muscle synergies, they have been frequently employed in rehabilitation scenarios. Relevant studies from Cheung and colleagues [18, 19] have studied how upper-limb spatial muscle synergies are organized in patients with different levels of impairment, in a repertoire of various upper-limb motions. While mildly impaired patients had physiological-like synergies, low functioning patients suffered from merging and/or fractionation issues, and the number of available modules was modified accordingly. It is indeed an open question whether robotic assistance may help to train proper coordination restoring the original number and composition of synergies, both during the interaction and as an effect of robotic therapies, and what categories of patients may find this approach beneficial. In fact, muscle synergies have the potential to become tools for clinicians to treat motor dysfunctions more effectively by organizing patients into subclasses and tailoring the treatment to each patient’s specific deficit [43].

Other studies have investigated a variety of upper-limb reaching movements and suggested that stroke induces abnormal coordination of muscle activation in severely impaired hemiparetic individuals by altering the structure of muscle synergies [44] or that alterations in the shoulder muscle synergies in stroke appear in an impairment level-dependent manner [45], and provided on overview of post-stroke patients affecting several aspects of the therapy such as prediction of outcomes, evaluation of the treatments, customization of doses, and therapies.

The works reported above show how muscle synergies were used as biomarkers of motor disability.

In the light of previous findings, we briefly discuss and suggest possible directions and applications for our results. The study presented protocols with increasing task complexity and showed that robust synergies are found in healthy subjects when switching across modalities. The achieved dataset, expanded appropriately, can be used to interpret patients’ neuro-motor coordination and evaluate his/her level of impairment based on reference data found on healthy subjects. Related to this, our findings open several research questions on intra-subject movement variability and differences in inter-subject motor coordination, especially when considering human-robot interaction which was less assessed than free movements in the muscle synergy framework. We noted that despite extracted synergies being very solid, some variability was observable on spatial synergies and on temporal coefficients. This finding might be related to the fact that subjects were measured in acute condition after a short adaptation period; it is possible that continuous training might lead to a further reduction of the motor variability. Variability was not evaluated in inter-session conditions; its quantification can be a crucial factor affecting the reliability of instrumental assessments in longitudinal studies where synergies are used as outcome measures [34]. Some kinematic/error variability was also found across directions and could be related to upper-limb manipulability ellipsoids defining limb dexterity in the upper-limb workspace [46]. This methodology, properly tuned depending on the application, could also be used to design evaluation protocols to be performed in free or robot-assisted movements. Future laboratory research may consider including higher movement variability and analyze in detail how it affects muscle synergies in the horizontal plane used for training. In clinical scenarios, experimenters might instead also consider subsets of the proposed tasks and movement directions (it is probably unrealistic to use the proposed paradigm in standard clinical use).

The results of this study may also find application on assistance paradigms. Depending on the correctness of the coordination obtained by the patient (measured by comparing patients’ patterns with free movement and reference healthy data), robotic assistance could be tuned according to an appropriate level of assistance, and even help clinicians to improve currently used assistance paradigms. This approach resembles the one proposed by Ferrante and colleagues [47] where typical muscle synergies from healthy people were used to determine patterns for functional electrical stimulation. Evaluation indexes might incorporate indexes related to the “naturalness” of the human-robot interaction, allowing to quantify motor patterns underlying movement. Synergy monitoring could be continuative and allow constant tuning of the robotic platform.

Lastly, we also wish to mention that the proposed approach is potentially scalable for the evaluation of human-robot interaction also in other fields of application, including non-planar assistive devices for rehabilitation, exoskeletons for rehabilitation and the industrial field.

### Limitations and future works

This work has some limitations. At first, it should be remarked that the use of 16 EMG channels, while being in line with the standard used in reference articles in the literature [37], is still limiting considering the huge number of motor units in the human upper-limb and trunk. Previous works warn about the use of a too small number of channels, suggesting that some modules might be missed or misinterpreted [48].

Secondly, while being comprehensive and inclusive of variability, the proposed mapping is still limited by the adoption of laboratory movements not yet integrated in real-life scenarios and realistic tasks. Boundary conditions related to interaction with objects or force application were not considered in this study. Furthermore, movements were performed at natural speed. A systematic evaluation of the effect of velocity was not performed.

Moreover, the presented analysis neglects the negative phasic EMG waveforms. While the range of applications may vary and reveal toward several fields, it is likely that, depending on the applications, the findings of this study might be further refined. Other methodological approaches (including synergy extraction algorithms and EMG normalization) could be considered to compare results. The application of this protocol to neurological patients’ performances might benefit from fine-tuned recordings for the matching of the reference database to the peculiar features of motor impairment (e.g.: reduced range of motion, jerky movements, lack of repeatability), which were not investigated in this study. The extracted synergies might be evaluated in the light of novel concepts such kinematic-muscular synergies to directly relate muscle activations to kinematic outputs.

Moreover, future work will make extensive use of kinematic, dynamic and EMG data for characterizing in more detail human-robot interaction and proposed multi-domain factorization algorithms.

The cohort of enrolled subjects is limited in number. Moreover, subjects could be grouped according to sex, age and height. Even exercises performed with the non-dominant limb could be analyzed and compared with those of the dominant limb.

Lastly, our results could be commented on in the light of the adoption of different assistance paradigms and performed tasks.

## Conclusions

In this paper, the effect of assistance provided with a robotic platform on healthy subjects was investigated with muscle synergies.

In our scenario involving a planar robotic device, we found that a limited number of synergies, correctly elicited by a control activation signal, underlies the execution of a large variety of rectilinear and curvilinear upper-limb movements when interacting with a robot device. Interestingly, while assistance influenced the adherence to the desired task, it influenced only marginally the motor control underlying movement. These findings can help a better understanding of human-robot interaction in the rehabilitative field.

## References

[1] Feigin VL. Global, regional, and national burden of neurological disorders, 1990–2016: a systematic analysis for the Global Burden of Disease Study 2016. Lancet Neurol. 2019. 10.1016/S1474-4422(18)30499-X PMC6459001 30879893

[2] WHO. Global Health Estimates 2015: Deaths by Cause, Age, Sex, by Country and by Region. 2000–2015. Glob. Heal. Estim. Tech. Pap.; 2017.

[3] Maciejasz P, Eschweiler J, Gerlach-Hahn K, Jansen-Troy A, Leonhardt S. A survey on robotic devices for upper limb rehabilitation. J Neuroeng Rehabil. 2014;11(1):3. doi: 10.1186/1743-0003-11-3 24401110 PMC4029785

[4] Zhang X, Yue Z, Wang J. Robotics in lower-limb rehabilitation after stroke. Behav Neurol. 2017;2017:1–13. doi: 10.1155/2017/3731802 28659660 PMC5480018

[5] Aisen ML, Krebs HI, Hogan N, McDowell F, Volpe BT. The effect of robot-assisted therapy and rehabilitative training on motor recovery following stroke. Arch Neurol. 1997;54(4):443–6. doi: 10.1001/archneur.1997.00550160075019 9109746

[6] Krebs HI, Ferraro M, Buerger SP, Newbery MJ, Makiyama A, Sandmann M, et al. Rehabilitation robotics: pilot trial of a spatial extension for MIT-Manus. J Neuroeng Rehabil. 2004;1(1):5. doi: 10.1186/1743-0003-1-5 15679916 PMC544952

[7] Riener R, Nef T, Colombo G. Robot-aided neurorehabilitation of the upper extremities. Med Biol Eng Comput. 2005;43(1):2–10. doi: 10.1007/BF02345116 15742713

[8] Volpe BT, Krebs HI, Hogan N, Edelstein OTR L, Diels C, Aisen M. A novel approach to stroke rehabilitation: robot-aided sensorimotor stimulation: Robot-aided sensorimotor stimulation. Neurology. 2000;54(10):1938–44. doi: 10.1212/wnl.54.10.1938 10822433

[9] Lunardini F, Casellato C, d’Avella A, Sanger TD, Pedrocchi A. Robustness and reliability of synergy-based myocontrol of a multiple degree of freedom robotic arm. IEEE Trans Neural Syst Rehabil Eng. 2016;24(9):940–50. doi: 10.1109/TNSRE.2015.2483375 26441423

[10] Reinkensmeyer DJ, Wolbrecht ET, Chan V, Chou C, Cramer SC, Bobrow JE. Comparison of 3D, Assist-as-Needed Robotic Arm/Hand Movement Training Provided with Pneu-WREX to Conventional Table Top Therapy Following Chronic Stroke. Am J Phys Med Rehabil. 2013. 10.1097/PHM.0b013e31826bce79 PMC3487467 23080039

[11] Just F, Özen Ö, Bösch P, Bobrovsky H, Klamroth-Marganska V, Riener R, et al. Exoskeleton transparency: feed-forward compensation vs. disturbance observer. at—Autom. 2018;66(12):1014–26.

[12] Abboudi RL, Glass CA, Newby NA, Flint JA, Craelius W. A biomimetic controller for a multifinger prosthesis. IEEE Trans Rehabil Eng. 1999;7(2):121–9. doi: 10.1109/86.769401 10391581

[13] Nam HS, Hong N, Cho M, Lee C, Seo HG, Kim S. Vision-assisted interactive human-in-the-loop distal upper limb rehabilitation robot and its clinical usability test. Appl Sci (Basel). 2019;9(15):3106.

[14] Yang J, Su H, Li Z, Ao D, Song R. Adaptive control with a fuzzy tuner for cable-based rehabilitation robot. Int J Control Autom Syst. 2016;14(3):865–75.

[15] Broekens J, Heerink M, Rosendal H. Assistive social robots in elderly care: a review. Gerontechnology. 2009. doi: 10.4017/gt.2009.08.04.004.00 25126043 PMC4130645

[16] Rodgers H, Bosomworth H, Krebs HI, van Wijck F, Howel D, Wilson N, et al. Robot assisted training for the upper limb after stroke (RATULS): a multicentre randomised controlled trial. Lancet. 2019;394(10192):51–62. doi: 10.1016/S0140-6736(19)31055-4 31128926 PMC6620612

[17] Bizzi E, Cheung VCK, d’Avella A, Saltiel P, Tresch M. Combining modules for movement. Brain Res Rev. 2008;57(1):125–33. doi: 10.1016/j.brainresrev.2007.08.004 18029291 PMC4295773

[18] Cheung VCK, Piron L, Agostini M, Silvoni S, Turolla A, Bizzi E. Stability of muscle synergies for voluntary actions after cortical stroke in humans. Proc Natl Acad Sci U S A. 2009;106(46):19563–8. doi: 10.1073/pnas.0910114106 19880747 PMC2780765

[19] Cheung VCK, Turolla A, Agostini M, Silvoni S, Bennis C, Kasi P. Muscle synergy patterns as physiological markers of motor cortical damage, 1–5. Proc Natl Acad Sci U S A. 2012;109(36):14652–14656. doi: 10.1073/pnas.1212056109 22908288 PMC3437897

[20] Lencioni T, Fornia L, Bowman T, Marzegan A, Caronni A, Turolla A, et al. A randomized controlled trial on the effects induced by robot-assisted and usual-care rehabilitation on upper limb muscle synergies in post-stroke subjects. Sci Rep. 2021;11(1):5323. doi: 10.1038/s41598-021-84536-8 33674675 PMC7935882

[21] Tropea P, Monaco V, Coscia M, Posteraro F, Micera S. Effects of early and intensive neuro-rehabilitative treatment on muscle synergies in acute post-stroke patients: a pilot study. J Neuroeng Rehabil. 2013;10(1):103. 24093623 10.1186/1743-0003-10-103 PMC3850948

[22] Scano A, Chiavenna A, Malosio M, Molinari Tosatti L, Molteni F. Robotic assistance for upper limbs may induce slight changes in motor modules compared with free movements in stroke survivors: A cluster-based muscle synergy analysis. Front Hum Neurosci. 2018;12:290. doi: 10.3389/fnhum.2018.00290 30174596 PMC6107841

[23] Yamine J, Prini A, Nicora ML, Dinon T, Giberti H, Malosio M. A planar parallel device for neurorehabilitation. Robotics. 2020;9(4):104.

[24] Chitta S, Marder-Eppstein E, Meeussen W, Pradeep V, Tsouroukdissian AR, Bohren J, et al. ros_control: A generic and simple control framework for ROS. Journal of Open Source Software. 2017;456(joss):00456.

[25] Dumas R, Chèze L, Verriest J-P. Adjustments to McConville et al. and Young et al. body segment inertial parameters. J Biomech. 2007;40(3):543–53. doi: 10.1016/j.jbiomech.2006.02.013 16616757

[26] Hermens HJ, Freriks B. SENIAM Project. The SENIAM project; 2017.

[27] Frère J, Hug F. Between-subject variability of muscle synergies during a complex motor skill. Front Comput Neurosci. 2012. doi: 10.3389/fncom.2012.00099 23293599 PMC3531715

[28] Scano A, Dardari L, Molteni F, Giberti H, Tosatti LM, d’Avella A. A comprehensive spatial mapping of muscle synergies in highly variable upper-limb movements of healthy subjects. Frontiers in physiology. 2019;10:1231. doi: 10.3389/fphys.2019.01231 31611812 PMC6777095

[29] d’Avella A, Portone A, Fernandez L, Lacquaniti F. Control of fast-reaching movements by muscle synergy combinations. J Neurosci. 2006;26(30):7791–810. doi: 10.1523/JNEUROSCI.0830-06.2006 16870725 PMC6674215

[30] Flanders M, Tillery SIH, Soechting JF. Early stages in a sensorimotor transformation. Behavioral and Brain Sciences. 1992;15(2):309–320.

[31] Rimini D., Agostini V., & Knaflitz M. Intra-subject consistency during locomotion: similarity in shared and subject-specific muscle synergies. Frontiers in human neuroscience. 2017; 11, 586. doi: 10.3389/fnhum.2017.00586 29255410 PMC5723022

[32] Oliveira AS, Gizzi L, Farina D, Kersting UG. Motor modules of human locomotion: influence of EMG averaging, concatenation, and number of step cycles. Frontiers in human neuroscience. 2014;8:335. doi: 10.3389/fnhum.2014.00335 24904375 PMC4033063

[33] Lee DD, Seung HS. Learning the parts of objects by non-negative matrix factorization. Nature. 1999. doi: 10.1038/44565 10548103

[34] Pale U, Atzori M, Müller H, Scano A. Variability of muscle synergies in hand grasps: Analysis of intra- and inter-session data. Sensors (Basel). 2020;20(15):4297. doi: 10.3390/s20154297 32752155 PMC7435387

[35] Eliana García-Cossio, Doris Broetz, Niels Birbaumer, Ander Ramos-Murguialday. Cortex integrity relevance in muscle synergies in severe chronic stroke. Frontiers in Human Neuroscience. 2014. 10.3389/fnhum.2014.00744 PMC4172028 25294998

[36] Coscia M, Cheung VCK, Tropea P, Koenig A, Monaco V, Bennis C, et al. The effect of arm weight support on upper limb muscle synergies during reaching movements. J Neuroeng Rehabil. 2014;11(1):22. doi: 10.1186/1743-0003-11-22 24594139 PMC3996016

[37] Pirondini E, Coscia M, Marcheschi S, Roas G, Salsedo F, Frisoli A, et al. Evaluation of the effects of the Arm Light Exoskeleton on movement execution and muscle activities: a pilot study on healthy subjects. J Neuroeng Rehabil. 2016;13(1):9. doi: 10.1186/s12984-016-0117-x 26801620 PMC4724067

[38] Pehlivan AU, Losey DP, OMalley MK. Minimal assist-as-needed controller for upper limb robotic rehabilitation. IEEE Trans Robot. 2016;32(1):113–24.

[39] Jarrasse N, Paik J, Pasqui V, Morel G. How can human motion prediction increase transparency? In: 2008 IEEE International Conference on Robotics and Automation. IEEE; 2008.

[40] Carmichael MG, Liu D. Experimental evaluation of a model-based assistance-as-needed paradigm using an assistive robot. Annu Int Conf IEEE Eng Med Biol Soc. 2013;2013:866–9. doi: 10.1109/EMBC.2013.6609638 24109825

[41] Morasso P, Casadio M, Giannoni P, Masia L, Sanguineti V, Squeri V, et al. Desirable features of a “humanoid” robot-therapist. Annu Int Conf IEEE Eng Med Biol Soc. 2009;2009:2418–21. doi: 10.1109/IEMBS.2009.5334954 19965200

[42] Moreno J. C., Barroso F., Farina D., Gizzi L., Santos C., Molinari M., et al. Effects of robotic guidance on the coordination of locomotion. Journal of neuroengineering and rehabilitation. 2013;10(1):1–15. doi: 10.1186/1743-0003-10-79 23870328 PMC3724716

[43] Jarque-Bou NJ, Sancho-Bru JL, Vergara M. A systematic review of EMG applications for the characterization of forearm and hand muscle activity during activities of daily living: Results, challenges, and open issues. Sensors (Basel) [Internet]. 2021;21(9). Available from: doi: 10.3390/s21093035 33925928 PMC8123433

[44] Roh J, Rymer WZ, Perreault EJ, Yoo SB, Beer RF. Alterations in upper limb muscle synergy structure in chronic stroke survivors. J Neurophysiol. 2013;109(3):768–81. doi: 10.1152/jn.00670.2012 23155178 PMC3567389

[45] Roh J, Rymer WZ, Beer RF. Evidence for altered upper extremity muscle synergies in chronic stroke survivors with mild and moderate impairment. Front Hum Neurosci. 2015;9:6. doi: 10.3389/fnhum.2015.00006 25717296 PMC4324145

[46] Jacquier-Bret J, Gorce P, Rezzoug N. The manipulability: a new index for quantifying movement capacities of upper extremity. Ergonomics. 2012;55(1):69–77. doi: 10.1080/00140139.2011.633176 22176485

[47] Ferrante S, Chia Bejarano N, Ambrosini E, Nardone A, Turcato AM, Monticone M, et al. A personalized multi-channel FES controller based on muscle synergies to support gait rehabilitation after stroke. Front Neurosci. 2016;10:425. doi: 10.3389/fnins.2016.00425 27695397 PMC5025903

[48] Steele KM, Tresch MC, Perreault EJ. The number and choice of muscles impact the results of muscle synergy analyses. Front Comput Neurosci. 2013;7:105. doi: 10.3389/fncom.2013.00105 23964232 PMC3737463

